# Are Trends in Economic Modeling of Pediatric Diabetes Mellitus up to Date with the Clinical Practice Guidelines and the Latest Scientific Findings?

**DOI:** 10.36469/001c.127920

**Published:** 2025-02-03

**Authors:** Roque Cardona-Hernandez, Alberto de la Cuadra-Grande, Julen Monje, María Echave, Itziar Oyagüez, María Álvarez, Isabel Leiva-Gea

**Affiliations:** 1 Division of Pediatric Endocrinology Hospital Sant Joan de Déu Barcelona; 2 Pharmacoeconomics and Outcomes Research Iberia (PORIB); 3 Health Economics & Outcomes Research Medtronic (Spain); 4 Pharmacoeconomics & Outcomes Research Iberia (PORIB); 5 Department of Pediatric Endocrinology Regional University Hospital of Malaga

**Keywords:** economic modeling, health technology assessment, literature review, pediatrics, type 1 diabetes, type 2 diabetes

## Abstract

**Background:** Modeling techniques in the field of pediatrics present unique challenges beyond traditional model limitations, and sometimes difficulties in faithfully simulating the condition’s evolution over time. **Objective:** This study aimed to identify whether economic modeling approaches in diabetes in pediatric patients align with the recommendations of clinical practice guidelines and the latest scientific evidence. **Methods:** A literature review was performed in March 2023 to identify modeling-based economic evaluations in diabetes in pediatric patients. Data were extracted and synthesized from eligible studies. Clinical practice guidelines for diabetes were gathered to compare their alignment with modeling strategies. Two endocrinology specialists provided insights on the latest findings in diabetes that are not yet included in the guidelines. A multidisciplinary group of experts agreed on the relevant themes to conduct the comparative analysis: parameter informing on glycemic control, diabetic ketoacidosis/hypoglycemia, C-peptide as prognostic biomarker, metabolic memory, age at diagnosis, socioeconomic status, pediatric-specific sources of risk equations, and pediatric-specific sources of utilities/disutilities. **Results:** Nineteen modeling-based studies (7 de novo, 12 predesigned models) and 34 guidelines were selected. Hemoglobin A1c was the main parameter to model the glycemic control; however, guidelines recommend the usage of complementary measures (eg, time in range) which are not included in economic models. Eight models included diabetic ketoacidosis (42.1%), 16 included hypoglycemia (84.2%), 2 included C-peptide (1 of those as prognostic factor) (10.5%) and 1 included legacy effect (5.3%). Neither guidelines nor models included recent findings, such as age at diagnosis or socioeconomic status, as prognostic factors. The lack of pediatric-specific sources for risk equations and utility/disutility values were additional limitations. **Discussion:** Economic models designed for assessing interventions in diabetes in pediatric patients should be based on pediatric-specific data and include novel adjuvant glucose-monitoring metrics and latest evidence on prognostic factors (C-peptide, legacy effect, age at diagnosis, socioeconomic status) to provide a more faithful reflection of the disease. **Conclusions:** Economic models represent useful tools to inform decision making. However, further research assessing the gaps is needed to enhance evidence-based health economic modeling that best represents reality.

## INTRODUCTION

Diabetes mellitus (DM) is a metabolic disease that leads to hyperglycemia due to defects in insulin secretion and/or function. In 2021, an estimated 537 million adults aged 20 to 79 years were living with diabetes worldwide, representing 10.5% of the world’s population in this age group.^1^ The total global number of people with diabetes is expected to increase to 643 million by 2030 and 783 million by 2045, representing 12.2% of the population.^1^ This poses a challenge for health systems because its high morbidity and mortality are linked to the onset of diabetic microvascular and macrovascular complications.^2,3^

The prevalence of type 1 diabetes (T1D) in children and adolescents is increasing.^4^ Although type 2 diabetes (T2D) remains rare in children,^5–7^ 201 000 new individuals younger than 20 years worldwide are estimated to develop T1D each year.^8^ In children and adolescents with T1D, biologically specific features, such as changes in daily insulin sensitivity patterns due to growth and sexual maturation processes or an inability to self-manage, should be considered.^9,10^ Moreover, neurological vulnerability to hypoglycemia and hyperglycemia, together with adverse neurocognitive effects on mental state, memory, and attention (all of which are associated with acute diabetic episodes),^11,12^ and the metabolic memory effects observed in patients with early-onset diabetes^13,14^ represent an additional risk for individuals who develop T1D during childhood and adolescence.

Given this burden, the development of novel therapeutics for children with T1D is increasing, driven by both pharmacological and technological interventions.^15–18^ In this context, economic evaluation of those novel therapies is key to inform decision making and thus ensuring the sustainability of healthcare systems and universal access to these treatments.^19–25^ Most of these economic evaluations are conducted by using modeling techniques.^26^

In pediatric populations, however, this exercise presents challenges beyond the traditional limitations associated with modeling to accurately simulate disease progression. Children and adolescents have different physiological and psychological needs than adults,^9–12^ and their long-term health outcomes can be significantly impacted by early interventions.^13,14^ Economic models tailored to the pediatric population must account for these unique factors so that healthcare providers can make more informed decisions that improve both immediate and long-term health outcomes for children with diabetes.

Therefore, modeling techniques in pediatric diabetes may sometimes present difficulties in faithfully simulating the evolution of the condition. This does not imply that models should not be used for decision making; rather, they offer valuable insights. However, the assumptions and evidence gaps regarding economic evaluations should be critically assessed.

In this sense, the evidence synthesis regarding methodological issues on economic evaluations provides an updated context on the state of the art by determining trends in disease modeling approaches, the availability of modeling techniques, and yields to assess the quality of those economic evaluations, all of which are key for guiding policy decisions based on economic evaluations.^27–29^ Literature reviews have focused on the features of economic models are available in the field of diabetes.^30–34^ However, none of these literature reviews are centered on pediatric patients.

Thus, the aim of this study was to identify whether economic modeling approaches for the evaluation of health interventions in patients with pediatric diabetes may be based on the recommendations of clinical practice guidelines and the latest scientific evidence in the field.

## METHODS

A literature review was performed to identify scientific publications about economic evaluations for pediatric diabetes. In addition, a manual search was conducted to retrieve the most updated clinical practice guidelines concerning diabetes.

A comparative analysis of the findings from these searches was performed to check the alignment between clinical key parameters considered in guidelines and those used when modeling DM. Considering the possibility that some recent scientific findings, including recent conclusions of clinical studies and/or therapeutic advances, could not be included yet in clinical practice guidelines, experts in the field of pediatric endocrinology were consulted to provide insight on these latest scientific findings and to validate the search strategy.

### Literature Review of Economic Models

The literature review was performed based on the Preferred Reporting Items for Systematic Reviews and Meta-Analyses (PRISMA) guidelines^35^ and recommendations for conducting literature reviews of economic evaluations.^27–29^

A PICO (Population-Intervention-Comparator-Outcome) question was defined based on the research question, “Which inputs are considered by the economic models designed for conducting both partial and complete economic evaluations of health interventions for pediatric diabetes?” The target population of the literature review consisted of pediatric patients with DM (≤25 years old, according to the endocrinologists’ criteria). Given that the aim of the study was to assess the modeling methods, the population was not restricted to any specific region. In addition, no restrictions were applied concerning the intervention, the comparator, or the outcomes.

Two strategies were designed by combining several terms using Boolean operators (OR/AND/NOT) (**Supplementary Tables S1** and **S2**). One strategy was designed to find both partial (studies aiming to estimate costs or health outcomes exclusively, eg, budget impact or cost-of-illness models) and complete (studies estimating the health outcomes achieved in relation to the costs, eg, cost-effectiveness, cost-utility, or cost-benefit models) economic evaluations performed with models, whereas the other aimed to identify systematic literature reviews (SLR) and/or meta-analyses (MA) related to the study objective.

The search was performed in Medline (through PubMed) on March 22, 2023. The identified studies were assessed for potential eligibility in a 2-phase procedure by 2 reviewers, who were also involved in the subsequent data extraction, quantitative synthesis of information, and qualitative assessment of the studies. First, the titles of the articles and abstracts were reviewed to evaluate whether they (1) aligned with the project objective and PICO question and (2) met the defined inclusion and exclusion criteria (**Supplementary Table S3**). Duplicates were also removed. In the second phase, the evaluators read the full texts of the selected studies to determine the eligible studies and collected the information of interest. Discrepancies between reviewers on whether to include or to exclude a study were resolved by a third reviewer.

The reference sections concerning publications about SLR and MA selected from the second search were reviewed to identify potential additional citations, which were added to those citations resulting from the first search, for full review and data extraction.

A data extraction matrix was developed in Microsoft Excel to synthetize the information from the eligible studies. The matrix included date of publication, country, type of diabetes, population, time horizon, intervention and comparators evaluated, type of economic evaluation, model characteristics (name, software selected for its design, structure and description of the model), efficacy measure and impact on outcomes, sources of efficacy and mortality, diabetic complications considered, sources of diabetic complications incidence, health utilities included, sources of health utilities, and measures of health outcomes. Information concerning the perspective of the analyses, costs included, year of costs, currency, discount rate, costs and outcomes results, and sensitivity analyses performed (including types and parameters modified) were not collected because these data were not related to the study objectives. The interventions evaluated were collected for characterizing the economic evaluation, not for analytic purpose regarding the aim of the study.

A qualitative analysis was performed on the extracted data. The models used in the studies identified were described, including their parameters, sources, and other information gathered. Patterns among models were assessed to provide aggregate data when feasible. Although no specific statistical plan was considered, descriptive statistics were estimated for quantitative variables to study potential trends. All these synthesized data were used in a subsequent comparative analysis of modeling techniques vs clinical practice guidelines and the latest scientific findings.

Furthermore, in line with the recommendations for literature reviews of economic evaluations,^27–29^ the International Society for Pharmacoeconomics and Outcomes Research (ISPOR)–Consolidated Health Economic Evaluation Reporting Standards (CHEERS) 2022 checklist was used to measure the methodological quality of the selected studies.^36^

### Search for Clinical Practice Guidelines

To find the latest clinical practice guidelines, a search in PubMed was also performed using the following terms in “All Fields”: “guideline,” “diabetes,” “pediatric,” “adolescents,” and “young adults.” This search was constrained to those clinical practice guidelines published in English or Spanish.

### Comparative Analysis

The multidisciplinary working group held a meeting to identify key themes of interest to compare economic models, clinical guidelines, and latest scientific evidence. The meeting was organized as a nominal group. Each expert proposed potential themes of interest and provided the basis for the proposal. Subsequently, the themes were selected when the agreement of all experts was reached.

The following selected themes comprised relevant scientific findings for the prognosis of diabetes in pediatric populations:

1. **Parameter informing on glycemic control:** Used in economic models to determine the risk of acute and chronic diabetic complications (eg, glycated HbA1c).
2. **Diabetic ketoacidosis (DKA) and hypoglycemic events:** Modeling DKA and/or hypoglycemia as diabetic complications that have an impact on health outcomes and costs.
3. **Evolution of the C-peptide as a prognostic biomarker:** To model in economic evaluations how C-peptide evolves in pediatric patients, in relation to insulin secretion.
4. **Metabolic memory or legacy effect:** To model in economic evaluations the influence of early and intense glycemic control during the patients’ lifetimes.
5. **Age at diabetes onset as potential endotype:** To model the different prognosis of the disease depending on the age when the diabetes onset was produced in each patient/cohort.
6. **Socioeconomic status of the patient’s family:** To model the socioeconomic status as a risk factor of worse glycemic control or diabetic complications onset.
7. **Pediatric-specific sources for risk equations:** This ensures that the risk equations used in modeling are based on studies relevant to the pediatric population, reflecting factors that specifically affect health outcomes and costs in children with diabetes. Characteristics of the clinical studies used to feed the inputs used to model the incidence of diabetic complications.
8. **Pediatric-specific sources for health utility/disutility values:** To model in economic evaluations the health utility/disutility values specific to the pediatric population, using data that reflect how diabetic complications affect the quality of life and well-being of pediatric patients.

The last theme was exclusive for complete economic evaluation concerning cost-utility analyses (CUAs).

The perspectives, interventions evaluated, and publication date were analyzed in relation to the model structure, quality, and alignment of the economic models with the latest scientific findings. Perspectives and interventions evaluated were assessed qualitatively. In the case of the year of publication, in addition to the qualitative analysis, descriptive statistics including range, median, interquartile range (IQR), mean, and standard deviation were estimated to evaluate potential trends. The aim of these estimates was to assess whether these variables were related to the inclusion of the themes described previously (eg, whether models published recently include DKA more frequently than models published a long time ago).

## RESULTS

### Literature Review Results: Economic Modeling Approaches

**Figure 1.**
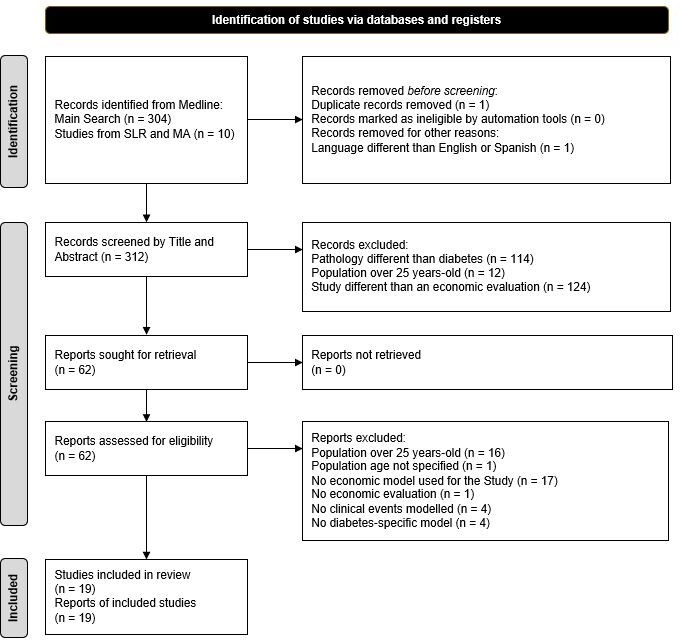
PRISMA Flowchart for Study Identification, Screening, and Inclusion Abbreviations: MA, meta-analysis; PRISMA, Preferred Reporting Items for Systematic Reviews and Meta-Analyses; SLR, systematic literature review.

The literature search resulted in 314 studies that were then assessed for eligibility (304 from the first strategy, and 10 from the 70 SLRs or MAs resulting from the second strategy) (**Supplementary Figure S1**). As shown in **Figure 1**, two studies were removed, one for being a duplicate and another for being written in Korean. The 312 remaining studies were screened according to their title and abstract content, leading to the exclusion of 250 more.

After a full review of the remaining 62 studies was carried out, 19 were ultimately selected for data extraction, and 43 publications were excluded. Studies that included patients without diabetes or patients older than 25 years (or nonreported age), economic evaluations performed without modeling (economic estimates provided by using real-world data from observational studies), those carried out with a non-diabetes-specific model (although the population consisted of children with diabetes, the aim of the economic evaluation was oriented to evaluate other events rather than diabetes, eg, cardiovascular disease in children with diabetes), or those excluding clinical events were removed. The general characteristics of the 19 partial or complete economic evaluations^37–55^ finally selected are summarized in **Table 1**.

**Table 1.** Characteristics of the Economic Evaluations Included in the Literature Review

**Reference**	**Country**	**Type of DM**	**Population (Age)**	**Intervention Evaluated**	**Comparators**	**Time Horizon**	**CHEERS 2022^36^ ^a^**	**Outcomes Measure**
Javitt (1990)^37^	US	T1D	Debutant patients (mean, 12.5 y)	Screening and treatment of ophthalmic diabetes complications	No screening strategy	Lifetime	24/26 items	Sight years saved/reading sight years saved/cost savings
Palmer (2000)^38^	Switzerland	T1D	19 y/o patients	Conventional treatment with insulin (Conv)	Conv + Retinopathy Screening (RS) /Conv + Microalbuminuria and iACE treatment (MS-iACE) /Conv + RS + MS-iACE/Intensive insulin treatment (Int) /Int + RS/Int + MS-iACE /Int + RS + MS-iACE	Lifetime	24/25 items	LYG
Roze (2005)^39^	UK	T1D	Adults^b^ (mean, 26 y/o; mean duration of DM: 12 y)	CSII	Insulin MDI	Lifetime	24/25 items	LYG and QALY
Cohen (2007)^40^	Australia	T1D	Adults^b^ and adolescents (mean, 17 y/o; mean duration of DM: 6 y)	CSII	Insulin MDI	60 y	24/25 items	LYG and QALY
Dall (2009)^41^	US	T1D, T2D	Patients of all ages	Not specified	^c^	Not specified	17/22 items	^c^
Gschwend (2009)^42^	Belgium, France, Germany, Italy and Spain	T1D	Patients 18-75 y/o	Detemir insulin	NPH + pre-prandial aspart insulin	50 y	19/25 items	QALY
Beckwith (2011)^43^	US	T1D	20 y/o patients with an acute hypoglycemic episode	Islet transplantation	Insulin therapy	20 y	23/25 items	QALY
Pfhol (2012)^44^	Germany	T1D	Adults^b^ (mean, 34.8 y/o; SD, 10 y)	Glargine insulin	Intensive NPH therapy	40 y	24/25 items	LYG and QALY
Gómez (2016)^45^	Colombia	T1D	Adults^b^ (mean, 34.19 y/o; SD, 17.14 y)	Insulin pump with a CGM device	Insulin MDI	55 y	24/25 items	LYG and QALY
Sussman (2016)^46^	US	T1D, T2D	Pediatric and adult patients in T1D/Adult patients in T2D	Insulin therapy with a technology that reduces risk of hypoglycemia	Insulin therapy without a technology that reduces the risk of hypoglycemia	1 y	22/25 items	Hypoglycemic episodes avoided
Dawoud (2017)^47^	UK	T1D	Adults^b^ (mean, 42.98 y/o; SD, 19.14 y)	Daily NPH	Daily detemir insulin /twice a day detemir insulin /Glargine insulin 100 IU /Degludec insulin /Twice a day NPH /4 times a day NPH	Lifetime	24/25 items	QALY
Roze (2017)^48^	Denmark	T1D	Adults with hyperglycemia at baseline^b^ (mean, 27.0 y/o; SD, 15.6 y) Adults with hypoglycemic risk at baseline^b^ (mean, 18.6 y/o; SD, 11.8 y)	Insulin SAP	CSII	Not specified	24/25 items	QALY
Thomas (2017)^49^	UK	T2D	Patients >16 y/o	Diabetes prevention program	No prevention program	20 y	24/25 items	LYG, QALY and social return of the investment of £1
GoodSmith (2019)^50^	US	T1D, T2D	Patients 10-20 y/o	MODY genes screening	No genetic screening	10 and 30 y	24/25 items	LYG and QALY
Roze (2019)^51^	Turkey	T1D	Adults with hyperglycemia at baseline^b^ (mean, 27.0 y/o; SD, 15.6 y) Adults with hypoglycemic risk at baseline^b^ (mean, 18.6 y/o; SD, 11.8 y)	Insulin SAP	CSII	Lifetime	24/25 items	QALY
McQueen (2020)^52^	US	T1D	Patients 2-17 y/o (mean: 9.3 y)	Autoimmune screening for kids and routine screening for diabetes	Habitual care in diabetes	Lifetime	23/25 items	QALY
Roze (2021)^53^	UK	T1D	Adult and adolescent patients (mean, 37.8 y/o; SD, 16.5 y)	Insulin pump	CSII	Lifetime	24/25 items	QALY
Pease (2022)^54^	Australia	T1D	12 y/o patients	CGM funded by the National Health System	CGM funded by the patient	9 y	24/25 items	QALY
Zhang (2022)^55^	China	T1D	Patients <18 y/o	CSII	MDI	70 y	23/25 items	LYG and QALY

Abbreviations: iACE, angiotensin convertor enzyme inhibitor; CGM, continuous glucose monitoring; CHEERS, Consolidated Health Economic Evaluation Reporting Standards; CSII, continuous subcutaneous insulin infusion; DM, diabetes mellitus; IU, International Units; LYG, life years gained; MDI, multiple daily injections; MODY, maturity-onset diabetes of the young; NPH, isophane insulin; QALY, quality-adjusted life year; SAP, sensor augmented pump; T1D, type 1 diabetes mellitus; T2D, type 2 diabetes mellitus; y/o, years old.

^a^ Number of items recorded in CHEERS 2022 checklist accomplished (**Supplementary Table S4**).

^b^ Adult population includes young adults (<25 years old).

^c^ The economic evaluation consisted of a cost analysis associated with T1D and T2D; thus, no intervention, comparators, or outcomes were described.

Concerning the economic evaluations identified in the literature review, 7 of the 19 studies (36.80%) were performed by developing novel models designed by the authors. Nevertheless, regarding diabetes, models have been developed by companies or healthcare institutions; these commercial models can be adapted by researchers with their own data. The predesigned models included the Core Diabetes Model (CDM) (9/19, 47.40%), the Cardiff Research Consortium (CRC) Discrete Event Simulation (DES) model (1/19, 5.25%), the School for Public Health Research (SPHR) Diabetes Prevention model (1/19, 5.25%), and the PROPHET model (1/19, 5.25%). Brief descriptions of the models are presented in **Table 2**.

**Table 2.** Description of the Economic Models Included in the Literature Review

**Model Name**	**References**	**Type of Model**	**Software**	**Model Description**
CDM^a^	^39,40,42,45,47,48,51,53,55^	Stochastic modeling via first-order Monte Carlo simulations	C++	The model cohort is first defined by age, sex, diabetes duration, racial characteristics, basal glycated HbA1c, blood pressure, BMI, lipidic profile, smoking habit, and basal complications. Complications considered included CVD (MI, angina, PVD, ictus, CHF, atrial fibrillation, and LVH), renal events (microalbuminuria, pronounced proteinuria, and ESRD), diabetic retinopathy (background retinopathy, proliferative retinopathy, and severe vision loss), macular edema, cataracts, foot ulcers (infected, gangrene, healed ulcerations and history of amputations), and diabetic neuropathy. The cohort demographics and presence or absence of complications determine the risk of new complications. The CDM includes 15 Markov submodels corresponding to each complication (including hypoglycemia, ketoacidosis, lactic acidosis, and nonspecific mortality). When a new complication develops, the patient transitions between health states of the complication-specific submodel were allowed, which were also influenced by the cohort demographics and presence of other complications. This design enables the existence of multiple complications in the same patient. All patient transitions occur in 1-year-long cycles. Changes in patient demographics derived from the intervention studied determine the risk of diabetic complications and, therefore, the LYG and QALYs. The CDM permits CEA, CUA and BIA.
CRC DES Model	^44^	DES^c^	Microsoft Excel® and C++	This model presents the same design as the CDM in terms of structure. However, instead of being programmed as a microsimulation (as in the case of the CDM), it was programmed as a DES.
PROPHET	^37^	Stochastic modeling via first-order Monte Carlo simulations^c^	^e^	Patients with T1D gain access to the model in the first year. The model simulates the patients’ transitions in 2-month cycles. First, the transition to death depends on the age at diagnosis and T1D-specific mortality. The surviving patients are at risk of disease progression (diabetic ophthalmic complications). Those patients whose disease progresses are screened. Progressing patients are identified according to the sensitivity and specificity of the screening. Patients who have ophthalmic complications according to the screening receive treatment to prevent the progression of T1D and mortality. The treatment efficacy is also modeled.
SPHR Diabetes Prevention Model	^49^	DES ^c^	^e^	This DES model is based on the individualized evolution of metabolic factors such as BMI, SBP, cholesterol, glycated HbA1c, etc. First, the model distributes the patients by age. Then, the patient’s metabolic factors are characterized, including the diagnosis of diabetes (determining basal glycated HbA1c level), during their first visit to a primary care physician. Second, patients are at risk of several comorbidities including CVD, diabetic complications (if their glycated HbA1c is >6.5%), cancer and skeletal complications. The presence of these comorbidities increases the risk of mortality. Finally, the risk of death for any cause is also considered. The process is repeated in 1-year cycles. The changes in the metabolic factors impact the risk of complications and the mortality.
De novo^b^	^38^	Deterministic Markov model^d^	^e^	The main Markov model includes renal, ophthalmic and CVD complications, in addition to hypoglycemic and ketoacidosis events. For each diabetic complication, there is a Markov sub-model programmed. The patients’ adherence to screening and subsequent treatment improves the control of clinical parameters, such as glycated HbA1c, which reduces the risk of diabetic complications.
	^41^	Cost calculator^d^	^e^	This tool allows for the estimation of patients with T1D and T2D and the proportion of patients who suffer several complications. Then, the respective costs are imputed.
	^43^	Deterministic Markov model^d^	Data TreeAge®	This Markov model includes 17 health states derived from the 7 more frequent complications in diabetes, including their pairwise combinations (representing the presence of 2 complications at the same time in the same patient). The diabetic complications considered are amputation (toe, foot, or leg), blindness (macular edema or diabetic retinopathy), CVD (nonlethal MI, silent MI, revascularization, angina and nonlethal ictus), ESRD, diabetic neuropathy, and death. The model was designed to study the transplantation of islet cells, which can cause PTLD in a proportion of patients, thus, this was accounted for in the cohort who received that intervention, leading to 33 health states. The effectiveness of the surgery affects the results of the economic evaluation. This effectiveness is measured by terms of the graft complete and partial function, or the graft failure based on the insulin requirements and C-peptide–detectable presence.
	^46^	Deterministic decision tree^d^	Microsoft Excel®	In this decision tree, the patients are divided in T1D, T2D, or nondiabetic. The T1D cohort is divided in 2 groups: ≥18 or <18 years old. All patients receive insulin treatment. The included patients with T2D must be ≥18 years old. Therefore, in this case, the group allocation depends on the insulin requirements of the patient. Every patient treated with insulin is at risk of hypoglycemia, which can be severe or mild-moderate. A decreased frequency of hypoglycemia affects the results of the economic evaluation.
	^50^	Stochastic decision tree and Markov model^d^	R	This decision tree begins by dividing the patients in 2 groups: the conventional management or genetic test groups. According to the prevalence, the patients in each arm are further classified in 4 groups: (1) MODY – GCK; (2) MODY – HNF-1A/HNF-4A; (3) T2D; and (4) T1D. In the conventional management arm, the patients receive a clinical diagnosis and subsequent treatment. In the genetic testing arm, patients coming from Groups 1 and 2 present autoAb and detectable C-peptide; thus, all patients are screened. The screening is used to determine whether that treatment is inappropriate for Group 1 patients (so these patients do not receive any treatment, optimizing the healthcare resource consumption). Additionally, the screening shows that the adequate therapy for Group 2 patients are sulfonylureas. For the patients allocated in Groups 3 and 4, the genetic test is performed only if the patient is autoAb negative, but C-peptide is detectable. The genetic test is used to determine the optimal pharmacotherapeutic strategy. All patient data are transferred to a Markov model including the data regarding diabetes complications (focusing on CVD complications). The level of glycated HbA1c after treatment is used to determine the risk of diabetic complications and therefore the results obtained in the economic evaluation.
	^52^	Deterministic Markov model^d^	Microsoft Excel®	This model is divided in 2 consecutive models. The first model simulates the screening of patients 2-18 years old with prediabetes, tracing them until their 30s. At age 30, the patients diagnosed with diabetes are transferred to a lifetime Markov model. This second model includes the health states of patients with neuropathy, retinopathy, ESRD, and CVD. The effectiveness of the early screening implies the reduction of the ketoacidosis, reducing the level of glycated HbA1c and, consequently, the incidence of diabetic complications.
	^54^	Deterministic Markov model^d^	Microsoft Excel® and Data TreeAge®	This Markov model is composed of several health states: T1D without complications, T1D with complications, and death. The diabetic complications considered include retinopathy, nephropathy, diabetic foot, acute MI, CHF and unstable chest angina. The differences between comparators are determined by their impact on glycemic HbA1c, which is related to the risk of complications. In addition, CGM systems are modeled to reduce the risk of hypoglycemic events.

Abbreviations: Ab, antibody; BIA, budget impact analysis; BMI, body mass index; CDM, core diabetes model; CEA, cost-effectiveness analysis; CGM, continuous glucose monitoring; CHF, congestive heart failure; CRC, Cardiff Research Consortium; CUA, cost-utility analysis; CVD, cardiovascular disease; DES, discrete event simulation; ESRD, end-stage renal disease; GCK, glucokinase; HNF, hepatic nuclear factor; LVH, left ventricle hypertrophy; LYG, life years gained; MI, myocardial infarction; MODY, mature-onset diabetes of the youth; PTLD, post-transplant lymphoproliferative disease; PVD, peripheral vascular disease; QALY, quality-adjusted life year; SBP, systolic blood pressure; SPHR, School for Public Health Research; T1D, type 1 diabetes mellitus; T2D, type 2 diabetes mellitus.

^a^ For detailed information about the CDM, see Palmer et al. *Curr Med Res Opin.* 2004;20(suppl 1):S5-26.

^b^ Noncommercialized economic model designed de novo for the study specific purposes.

^c^ Patient-level simulation model.

^d^ Cohort-level simulation model.

^e^ Data not available.

Regardless of whether the models were de novo or predesigned, the economic models developed for the evaluation of interventions for pediatric diabetes matched their approach, which consisted of modeling the development of diabetic complications.

The frequency of diabetic complications was determined by several strategies. The relationship with the glycated hemoglobin (HbA1c) level was the preferred parameter included in the models. Ten of the 12 economic evaluations including HbA1c (83.30%) considered the influence of other factors on the incidence of diabetes complications, such as body mass index (BMI), lipid profile, sex, smoking habit, blood pressure, diabetes onset, or age. All these parameters were included in several risk equations, which determined the risk of each of the diabetic complications considered.

The data sources used for establishing the relationship between these factors and the incidence of complications included studies such as the Diabetes Control and Complications Trial (DCCT)^56^ and the Epidemiology of Diabetes Interventions and Complications (EDIC)^57^ in populations with T1D, or the United Kingdom Prospective Diabetes Study (UKPDS)^58,59^ in populations with T2D, in addition to other studies with large sample sizes such as the Framingham Study.^60,61^ The efficacy of the evaluated interventions was essentially determined through clinical trials. Other strategies directly modeled the incidence of complications for each comparator.

### Recommendations from the Clinical Practice Guidelines

A total of 34 guidelines were reviewed (**Supplementary Table S5**): 1 from Australia, 1 from China, 1 from Korea, 1 from Canada, 7 from the United States, 1 from Germany, 1 from Italy, 6 from Spain, 4 from the United Kingdom, and 6 from several European countries. The remaining 5 guidelines were published as a result of an international collaboration. In summary, the main clinical parameters recommended by the guidelines to be considered in clinical practice and their target values recommended for any treatment are summarized in **Table 3**. Other recommendations focused on patient-dependent parameters (hypoglycemic risk, kidney function, cardiovascular risk, age, and sex), treatment characteristics (adverse effects or treatment complexity), or available healthcare resources.

**Table 3.** Clinical Practice Guidelines Recommendations

	**American Guidelines**	**Australasian Guidelines**	**European Guidelines**	**International Guidelines**
	Canada: Government US: ADA, AACE, AAP	APEG China: DAROC Korea: KD	EASD Germany: DDG Italy: SIEDP Spain: SED and Government UK: NICE and Government	Collaborations between societies ISPAD
Glycated HbA1c	<7% (53 mmol/mol) An HbA1c level >8% could be considered in patients with high risk of hypoglycemia	<6.5% (48 mmol/mol)/<7% (53 mmol/mol)	<6.5% (48 mmol/mol)/<7% (53 mmol/mol) <7.5% (58 mmol/mol) in patients <18 years old An HbA1c level <8% could be considered in patients with high risk of hypoglycemia	<7% (53 mmol/mol) An HbA1c level <6.5% or <7.5% could be consider in several context
Preprandial glucose	90-130 mg/dL (5.0-7.2 mmol/L)	80-130 mg/dL (4.5-7.2 mmol/L)	<125 mg/dL A glucose level <140 mg/dL could be considered in patients with high risk of hypoglycemia	70-145 mg/dL (4.0-8.0 mmol/L)
Postprandial glucose	–	80-160 mg/dL (4.5-8.9 mmol/L)	<180 mg/dL (>10.0 mmol/L) A glucose level under 140 mg/dL is considered the optimal target level A glucose level <200 mg/dL could be considered in patients with high risk of hypoglycemia	90-180 mg/dL (5.0-10.0 mmol/L)
GMI ^a^	70-180 mg/dL (3.9-10.0 mmol/L)	–	–	–
Definition of TIR	70-180 mg/dL (3.9-10.0 mmol/L)	–	70-180 mg/dL (3.9-10.0 mmol/L)	70-180 mg/dL (3.9-10.0 mmol/L)
Target TIR	TIR ≥70%	–	TIR ≥70%	-
Definition of TAR	Level 1: >181-250 mg/dL (10-13.9 mmol/L) Level 2: >250 mg/dL (>13.9 mmol/L)	–	Level 1: >181-250 mg/dL (10-13.9 mmol/L) Level 2: >250 mg/dL (>13.9 mmol/L)	Level 1: >181-250 mg/dL (10-13.9 mmol/L) Level 2: >250 mg/dL (>13.9 mmol/L)
Target TAR	–	–	TAR Level 1 <25% TAR Level 2 <5%	-
Definition of TBR	Level 1: 54-69 mg/dL (3.0-3.9 mmol/L) Level 2: <54 mg/dL (<3.0 mmol/L)	–	Level 1: 54-69 mg/dL (3.0-3.9 mmol/L) Level 2: <54 mg/dL (<3.0 mmol/L)	Level 1: 54-69 mg/dL (3.0-3.9 mmol/L) Level 2: <54 mg/dL (<3.0 mmol/L)
Target TBR	TBR <4% TBR Level 2 <1% A TBR Level 2 >1% could be consider in patients with high risk of hypoglycemia	–	TBR <4% TBR Level 2 <1%	–
Glucose variation coefficient	≤36%	–	<36%	–
Weight reduction	3-7% is considered acceptable, 10% is considered optimal	>5% Waist circumference <90 cm (men) or 80 (women) BMI 18.5-24 kg/m^2^	–	–
Caloric expenditure	≥ 700 kCal /week		–	–
Blood pressure	SBP <130 mmHg SBP should be reduced to 115-135 in pregnant patients DBP <80 mmHg DBP should be reduced <85 in pregnant patients	If cardiovascular disease is present: SBP <140 /130 mmHg DBP <90/80 mmHg If cardiovascular disease is absent: SBP <140 mmHg DBP <85 mmHg	–	–
Lipidic profile	Reduction of LDL-C ≥ 50% reaching a level <70 mg/dL In patients with established atherosclerosis, it should be considered to reach a level of LDL-C under 55 mg/dL	If cardiovascular disease is present: LDL-C <70 mg/dL HDL-C >40 mg/dL (men)/50 mg/dL (women) Triglycerides <150 mg/dL If cardiovascular disease is absent: LDL-C <100 mg/dL HDL-C >40 mg/dL (men)/50 mg/dL (women) Triglycerides <150 mg/dL	–	LDL-C <100 mg/dL HDL-C >35 mg/dL Triglycerides <150 mg/dL
GFR	>20 mL/min/1.73 m^2^	–	–	–
Urinary albumin	>200 mg/g	–	–	–

Abbreviations: AACE, American Association of Clinical Endocrinology; AAP, American Association for Pediatrics; ADA, American Diabetes Association; APEG, Australasian Paediatric Endocrine Group; BMI, body mass index; DBP, diastolic blood pressure; DAROC, Diabetes Association of the Republic of China (Taiwan); DDG, Deutsche Diabetes Gesellschaft (Germany Diabetes Association); EASD, European Association for the Study of Diabetes; GFR, glucose filtration rate; GMI, Glucose Management Index; ISPAD, International Society for Pediatric and Adolescent Diabetes; KDA, Korean Diabetes Association; LDL-C, low density lipoprotein cholesterol; NICE, National Institute for Health and Care Excellence; SBP, systolic blood pressure; SED, Sociedad Española de Diabetes (Spanish Diabetes Society); SIEDP, Società Italiana Endocrinologia e Diabetologia Pediatrica (Italian Society for Pediatric Endocrinology and Diabetology); TAR, time above range; TBR, Time below range; TIR, time in range.

^a^ GMI is defined as the glucose concentration measured during 24 hours by continuous glucose monitoring systems.

### Comparative Analysis: Economic Models vs Latest Scientific Findings in Pediatric Diabetes

The comparative analysis revealed that, given that most economic evaluations were conducted by using predesigned models, neither the perspectives, interventions evaluated, nor date of publication showed any relationship with the model structure, quality, and alignment of the studies with the latest scientific findings.

The detailed analysis for all themes of comparison was not feasible due to the low number of models, including each scientific finding. Thus, it is provided only for the theme concerning the inclusion of DKA and hypoglycemic events. The results of the comparative analysis are described below and summarized in **Table 4**, which includes the rationale for including the theme and considerations of the experts in relation to the topic.

**Table 4.** Summary of the Comparative Analysis Results

**Themed Category**		**Rationale for Inclusion as a Theme**	**Inclusion of the Theme**		**Experts’ Considerations**
			**Economic Evaluations**	**Clinical Practice Guidelines**	
1	Parameters informing on glycemic control	Novel glycemic metrics complementary to the HbA1c have been proposed since the usage of CGM devices	HbA1c	HbA1c, TIR, TAR, TBR and glycemic variability coefficient	Further research would be necessary to better stablish the relationship between those novel metrics and the risk of diabetic complications
2	DKA and hypoglycemic events	DKA and hypoglycemia are especially relevant in the pediatric setting according to the experts’ criteria	Inclusion of DKA: 8/19 studies (42.1%) Inclusion of hypoglycemia: 16/19 studies (84.2%)	Recommendation for a close monitoring in children	DKA and hypoglycemia should be modeled as the available evidence makes it feasible
3	Evolution of the C-peptide as a prognostic biomarker	C-peptide is surrogate parameter reflecting a decrease in insulin secretion^62,63^	1/19 (5.3%)	Not included	Further research would be necessary to better define the prognostic value of the C-peptide
4	Metabolic memory or legacy effect	Glycemic control in childhood/adolescence predicts metabolic control in early adulthood^64,65^	1/19 (5.3%)	Included in ADA 2023 Standards of Care^66^	Further research would be necessary to better define the prognostic value of the legacy effect
5	Age at diabetes onset (potential endotype)	The endotype may be related to the glycemic, genetic, or metabolic disease profile and may eventually be related to a decrease in beta cell count and disease prognosis^67–69^	Not included	Not included	Further research would be necessary to better define the prognostic value of the endotype
6	Socioeconomic status of the patient’s family	The socioeconomic status of parents and caregivers may be a determining factor for the achievement of glycemic goals^70,71^	Not included	Not included	Further research would be necessary to better define the prognostic value of the socioeconomic status of the patients’ family
7	Pediatric-specific sources for risk equations	Potential lack of studies specific to the pediatric population with diabetes according to the experts’ criteria	The main sources (DCCT, EDIC and UKPDS) do not represent the pediatric population well	–	Further research would be necessary to define risk equations for diabetic complications specific to the pediatric setting
8	Pediatric-specific sources for the utility/disutility values	Potential lack of studies specific to the pediatric population with diabetes according to the experts’ criteria	The sources of health utility/disutility values do not include populations aligned with the target pediatric patients included in the models	–	Further research would be necessary to define health utility/disutility values specific to the pediatric setting

Abbreviations: ADA, American Diabetes Association; CGM, continuous glucose monitoring; DCCT, Diabetes Control and Complications Trial; DKA, diabetic ketoacidosis; EDIC, Epidemiology of Diabetes Interventions and Complications; TAR, time above range; TBR, time below range; TIR, time in range; UKPDS, United Kingdom Prospective Diabetes Study.

**Parameters informing on glycemic control**: In the case of pediatric diabetes, the economic models included HbA1c, the main parameter that determines the risk of diabetes complications according to the latest scientific evidence included in clinical practice guidelines.

However, the clinical practice guidelines also recommended the use of complementary parameters, including time in range (TIR), time above range (TAR), time below range (TBR), and glycemic variability coefficient. All these novel metrics are still not considered in any model used for economic evaluation of interventions in pediatric diabetes.

**DKA and hypoglycemic events**: DKA and hypoglycemia episodes were the least common complications incorporated in economic models. Eight economic evaluations exclusively modeled DKA events (42.1%), and 16 included hypoglycemic events (84.2%).

The models including DKA were published between 2000 and 2022. The median year of publication was 2014.5 (IQR, 2008-2019.25); the mean year was 2013 (SD, 7.86). Compared with those models not including DKA events (range, 1990-2022; median, 2016; IQR, 2010-2018; mean, 2013; SD, 9.03), these descriptive statistics revealed no relationship between the date of publication and the inclusion of DKA events. The studies including DKA events evaluated screening strategies and insulin regimens. In contrast, a notably greater number of studies evaluating devices for continuous glucose monitoring (CGM) were found between the economic evaluations excluding DKA events, which might be indicative of a trend. No trends were observed in relation to the perspective of the analyses.

In the case of hypoglycemia, heterogeneity was observed in terms of how it was modeled. Some models included hypoglycemia as a single event, instead of as a model health state. Similarly, differences were also observed if developers opted to enable a transition from hypoglycemia to death. Regarding the influence of the date of publication, studies including hypoglycemic events were published between 2000 and 2022 (median, 2016.5; IQR, 2010.5-2019.25; mean, 2015; SD, 6.57). Differences in the publication periods were thought to be caused by the limited number of studies excluding these events (range, 1990-2017; median, 2009; IQR, 1999.5-2013; mean, 2005; SD, 13.87). Similarly, no remarkable trends were found for the influence of perspectives and interventions evaluated.

**Evolution of the C-peptide as a prognostic biomarker**: Two economic evaluations modeled C-peptide levels. One included this parameter as a determinant of islet transplantation failure,^3^ and the other used C-peptide levels as a screening strategy to detect monogenic diabetes genes.^50^

**Metabolic memory or legacy effect**: Although the American Diabetes Association (ADA) 2023 Standards of Care recommends strict management of diabetes in childhood and adolescence early after diagnosis,^66^ the legacy effect was modeled in just 1 economic evaluation among those identified.^52^

**Age at diabetes onset as potential endotype**: The age at diabetes onset, which could be an endotype,^67^ was not modeled in any of the identified economic evaluations.

**Socioeconomic status of the patient’s family**: The socioeconomic status of the patient’s family was not modeled in any of the identified economic evaluations.

**Pediatric-specific sources for the risk equations**: Most models used data from DCCT,^56^ EDIC,^57^ and UKPDS^58,59^ to determine the risk equation for the several complications modeled. In the DCCT, patients aged 13 to 39 years old were enrolled,^56^ most of whom were later recruited for the EDIC study.^57^ The participants in the UKPDS were aged 25 to 65 years.^58,59^ Thus, the pediatric population modeled in economic evaluations is not well represented in those epidemiological studies.

**Pediatric-specific sources for the utility/disutility values**: Fifteen studies (78.9%) included a CUA using utility and/or disutility values on their models, with up to 50 references cited (**Supplementary Table S5**), which were subsequently analyzed (**Table 5**). Among the 50 sources cited by the economic evaluations involving CUAs, a unique study providing utilities and disutilities was designed specifically for patients under 18 years of age.^72^ Twelve additional studies enrolled patients younger than 25.

Moreover, as observed in **Table 5**, discrepancies can be found between the type of diabetes of the target population of the studies providing utility/disutility values and the modeling-based CUAs. Among the 3 most cited sources, the utility values estimated by Beaudet et al were applied in 6 economic evaluations of pediatric participants with T1D, but the population enrolled in the study comprised individuals with T2D.^73^ In Clarke et al, the values were obtained from an adult population with T2D,^74^ which does not fit the population considered in the economic analyses (5 focused on T1D while 1 included a cohort of participants with T1D and T2D). Likewise, Currie et al studied the health-related quality of life (HRQoL) in a sample of adult patients with either T1D or T2D.^75^ In this case, the economic evaluations using that source of utility/disutility values targeted pediatric individuals with T1D; therefore, they used utility values that did not fit the target population due to a lack of pediatric-specific data.

**Table 5.** Sources for Utility and Disutility Values Most Cited

**Sources ^a^**	**Health State Utility/Disutility Source Characteristics**					**Characteristics of the Economic Evaluations Referencing the Source**			
	**Target Population/ Disease**	**Country**	**HRQoL Survey**	**Type of Study**	**Includes Pediatric Population**	**Economic Evaluations Referencing the Source (N = 15)**	**Target Population**		
							**T1D**	**T2D**	**T1D/T2D**
Beaudet (2014)	T2D	UK	EQ-5D	Clinical study	No	6 (40.00%)	6/6	0/6	0/6
Clarke (2002)	T2D	UK	EQ-5D	Clinical study	No	6 (40.00%)	5/6	0/6	1/6
Currie (2006)	T1D/T2D	UK	EQ-5D	Clinical study	No	6 (40.00%)	6/6	0/6	0/6
Tengs (2000)	^b^	^b^	^b^	Modeling study	^b^	4 (26.70%)	4/4	0/4	0/4
Nørgaard (2013)	T1D/T2D	Europe ^C^ and Israel	HFS	Clinical study	Yes (28 ± 15.7 years old)	3 (20.00%)	3/3	0/3	0/3
AIHW (2003)		Australia	^b^	Clinical study	^b^	2 (13.35%)	2/2	0/2	0/2
Carrington (1996)		UK	HAD	Clinical study	No	2 (13.35%)	2/2	0/2	0/2
Harris (2014)	T1D/T2D	Canada	TTO study	Clinical study	No	2 (13.35%)	1/2	0/2	1/2
McBride (2013)	T1D	Australia	EQ-5D	Clinical study	Yes (23.2 ± 11.9 years old)	2 (13.35%)	2/2	0/2	0/2
Nørgaard (2012)	T1D/T2D	Europe ^c^ and Israel	HFS	Clinical study	^b^	2 (13.35%)	2/2	0/2	0/2
Palmer (2004)	T1D/T2D	UK	^b^	Economic evaluation	^b^	2 (13.35%)	1/2	0/2	1/2
Alva (2013)	T2D	UK	EQ-5D	Clinical study	No	1 (6.67%)	0/1	1/1	0/1
Bagust (2005)	T2D	Belgium, Spain, Italy, Netherlands and Sweden	EQ-5D	Clinical study	No	1 (6.67%)	1/1	0/1	0/1
Begg (2007)	^b^	Australia	^b^	Clinical study	^b^	1 (6.67%)	0/1	0/1	1/1
Benedict (2010)	Depression	Europe	EQ-5D	Clinical study	^b^	1 (6.67%)	0/1	1/1	0/1
Black (2009)	General population	Multinational	WOMAC/HUI	SLR	No	1 (6.67%)	0/1	1/1	0/1
CD (2010)	T1D/T2D	^b^	^b^	Economic evaluation	^b^	1 (6.67%)	1/1	0/1	0/1
Cheng (2017)	T1D/T2D	Netherlands	TTO study	Clinical study	Yes (inclusion criteria: 17-70 years)	1 (6.67%)	1/1	0/1	0/1
Clarke (2004)	T2D	UK	EQ-5D	Clinical study	No	1 (6.67%)	1/1	0/1	0/1
Coffey (2002)	T1D/T2D	USA	EQ-5D	Clinical study	No	1 (6.67%)	0/1	1/1	0/1
Colagiuri (2009)	^b^	Australia	^b^	Clinical study	^b^	1 (6.67%)	1/1	0/1	0/1
Dolan (1995)	General population	UK	EQ-5D	Clinical study	No	1 (6.67%)	0/1	1/1	0/1
Evans (2013)	T1D/T2D	Sweden, Germany, Canada, UK and USA	TTO study	Clinical study	No	1 (6.67%)	1/1	0/1	0/1
Goldney (2004)	T1D/T2D	Australia	SF-36	Clinical study	Yes (inclusion criteria: ≥15 years)	1 (6.67%)	1/1	0/1	0/1
Hiratsuka (2013)	Cataract	Japan	EQ-5D/HUI	Economic evaluation	No	1 (6.67%)	1/1	0/1	0/1
Kawasaki (2015)	T1D/T2D	Japan	TTO study	Economic evaluation	No	1 (6.67%)	1/1	0/1	0/1
Lee (2005)	Renal failure	UK	EQ-5D/SF-36/KDQOL	Clinical study	No	1 (6.67%)	1/1	0/1	0/1
Lee (2011)	T1D	UK	HUI	Clinical study	Yes ^d^	1 (6.67%)	0/1	0/1	1/1
Matza (2007)	T2D	UK	EQ-5D/PGWB/ADS	Clinical study	No	1 (6.67%)	1/1	0/1	0/1
McEwan (2007)	T2D	UK	^b^	Economic evaluation	No	1 (6.67%)	1/1	0/1	0/1
McQueen (2014)	T1D	USA	EQ-5D	Clinical study	No	1 (6.67%)	1/1	0/1	0/1
Morgan (2006)	T1D/T2D	UK	EQ-5D	Clinical study	Yes (inclusion criteria: ≥15 years)	1 (6.67%)	1/1	0/1	0/1
NHS (2010)	^b^	UK	^b^	^b^	^b^	1 (6.67%)	0/1	0/1	1/1
NICE (2015)	^b^	UK	^b^	^b^	^b^	1 (6.67%)	1/1	0/1	0/1
NICE (2015)	^b^	UK	^b^	^b^	^b^	1 (6.67%)	0/1	0/1	1/1
NICE (2002)	^b^	UK	^b^	^b^	^b^	1 (6.67%)	1/1	0/1	0/1
Nyman (2007)	T1D/T2D	USA	EQ-5D	Clinical study	Yes (inclusion criteria: ≥18 years)	1 (6.67%)	1/1	0/1	0/1
Pratoomsoot (2009)	T1D ^E^	UK	EQ-5D	Economic evaluation	No	1 (6.67%)	1/1	0/1	0/1
Redekop (2004)	T1D/T2D	Netherlands	TTO study	Clinical study	Yes (inclusion criteria: 17-70 years)	1 (6.67%)	1/1	0/1	0/1
Smith-Palmer (2016)	T1D	^f^	EQ-5D/15-D/QWBS/TTO study/SG	SLR	No	1 (6.67%)	1/1	0/1	0/1
Sullivan (2006)	T1D/T2D	USA	EQ-5D	Clinical study	Yes (inclusion criteria: ≥18 years)	1 (6.67%)	1/1	0/1	0/1
Szabo (2010)	T1D/T2D	USA and Canada	TTO study	Clinical study	No	1 (6.67%)	1/1	0/1	0/1
Tabaei (2004)	T1D/T2D	USA	QWBS	Clinical study	Yes (inclusion criteria: ≥18 years)	1 (6.67%)	1/1	0/1	0/1
USGAO (2007)	Kidney disease	USA	^b^	Clinical study	Yes (inclusion criteria: ≥18 years)	1 (6.67%)	1/1	0/1	0/1
Walters (2006)	T1D	Australia	HFS	Clinical study	No	1 (6.67%)	0/1	0/1	1/1
Ward (2007)	Coronary disease	^b^	^b^	SLR	^b^	1 (6.67%)	0/1	1/1	0/1
Wasserfallen (2004)	Dialysis	Switzerland	EQ-5D	Clinical study	No	1 (6.67%)	1/1	0/1	0/1
Wolowacz (2015)	T1D	^b^	^b^	Economic evaluation	^b^	1 (6.67%)	0/1	0/1	1/1
Yabroff (2004)	Cancer	UK	HALex	Clinical study	Yes (inclusion criteria unrestricted to age)	1 (6.67%)	0/1	1/1	0/1
Yeh (2012)	^b^	^b^	^b^	SLR	^b^	1 (6.67%)	1/1	0/1	0/1

Abbreviations: ADS, Appraisal of Diabetes Scale; AIHW, Australian Institute of Health and Welfare; EQ-5D, EuroQol 5 Dimensions; HAD, Hospital Anxiety and Depression; HALex, Health Activities and Limitations Index; HFS, Hypoglycemia Fear Survey; HRQoL, health-related quality of life; HUI, Health Utility Index; KDQOL, Kidney Disease Quality of Life; NHS, National Health System; NICE, National Institute for Health and Care Excellence; PGWB, Psychological General Well-Being Index; QWBS, Quality of Well-Being Scale; SF-36, Short-Form 36 items; SG, Standard Gamble; SLR, Systematic Literature Review; TTO, time trade-off; T1D, type 1 diabetes mellitus; T2D, type 2 diabetes mellitus; USGAO, United States Government Accountability Office; 15-D, 15-Dimensional.

^a^ The references of these sources are presented in **Supplementary Table S6**.

^b^ Data not available.

^c^ The study included 15 European countries.

^d^ Study specifically designed to measure the HRQoL of pediatric patients with T1D.

^e^ The economic evaluation used data from patients with T2D enrolled in the UKPDS for patients with T1D.

^f^ Wide variety of countries/HRQoL surveys identified in the SLR.

## DISCUSSION

Economic evaluations based on modeling techniques provide key information for decision making. Economic models present assumptions and lack of information. Thus, it is important to critically evaluate these issues in order to better inform decision makers, who might rely on additional sources of evidence in combination to model-based studies (eg, real-world evidence studies, clinical trials, etc.) to provide a robust basis to their decisions.

Previous attempts to synthesize the methodology for conducting modeling-based economic evaluations are available in the field of diabetes.^30–34^ The most recent literature review on the modeling strategies for diabetes was not focused on a specific population.^34^ According to the study, despite their programming complexity, patient-level models were found to be widely used in diabetes due to their flexibility, as opposed to cohort models. These models, which are designed to provide evidence for decision makers, were considered high quality. In addition, the authors of the aforementioned SLR stated that methods used for modeling represent modern and evolving state-of-the-art features.^34^ These conclusions were consistent with similar studies focused specifically on T1D^31^ and T2D.^32^ In addition, a review of the models used by the National Institute for Health and Care Excellence (NICE) to assess treatments in both T1D and T2D stated that those models are helpful instruments for decision making, but they are not exempt from methodological issues that should be addressed; thus, additional evidence should be taken into account to support these models’ findings.^33^

In this sense, the differences between adults and pediatric populations result in the need to consider additional inputs when modeling the evolution of the disease in this young population.^9–14^ Given that many models in pediatric diabetes are encompassed between the models used for adult populations, some of these findings provided by other authors could be applicable to those models in pediatric diabetes. However, to provide an accurate picture of the modeling strategies in pediatric diabetes, those specific issues derived from the target population should be assessed^9–14^; this is the main motivation to conduct this study.

To our knowledge, this is the first study to critically assess the modeling strategies developed to evaluate interventions for pediatric patients with diabetes, presenting a comprehensive analysis of the latest scientific findings in the field, and thus highlighting potential areas for improvement in the development of these tools. The final objective of this study is to strengthen the modeling approaches used in this field in order to achieve the most faithful reflection of the disease based on the latest available scientific evidence to support decision making.

Given the latest findings in pediatric diabetes, 8 themes of interest were found to be especially relevant to include in models developed for this population.

First, the use of diabetes technology tools, such as CGM, has led scientific societies to consider new parameters to assess diabetes management. These novel parameters include the TIR, TAR, TBR, and glycemic variability coefficient. The HbA1c remains the primary outcome measure for assessing glycemic control as it is a surrogate marker for long-term diabetes complications; however, it has several limitations: (1) it may not accurately reflect average glucose levels, as a wide range of mean glucose concentrations and glucose profiles can be associated with a certain HbA1c level, and it can be altered by several factors other than glucose,^76,77^ and (2) it cannot be used to predict the frequency and/or risk of experiencing hyperglycemia or hypoglycemia.^78–80^ For both of these reasons, TIR is recommended by international clinical societies as an adjuvant measure to HbA1c to provide a complete description of the glycemic profile. Using TIR may have several advantages in assessing glycemic control because (1) TIR is a measure of glycemic dispersion,^81,82^ (2) it is a key metric of glucose management that includes short-term glycemic variations along with the risk of experiencing acute complications,^81,83^ and (3) it may be helpful to individualize a therapeutic plan.^82,84^ Nevertheless, there is limited evidence about the relationship between changes in these new CGM-based metrics and variations in HbA1c.^85^ Thus, modeling a potential reduction in diabetic complications based on changes in the TIR, TAR, and TBR is not possible to date. Future research strategies are needed to clarify these relationships, which could improve the accuracy of economic evaluations in the pediatric diabetes setting.

Second, hypoglycemic and DKA events are key complications to be considered in modeling studies simulating pediatric patients. Although the inclusion of hypoglycemia is extended in the economic models, the inclusion of DKA remains rare. It should be noted that many of the economic evaluations identified compared CGM devices; thus, the exclusion of DKA in the models evaluating those medical devices could be based on the reduced rate of these acute events associated with CGM devices. In any case, the modeling of both DKA and hypoglycemic events should be considered more frequently in economic evaluations targeted to pediatric patients with diabetes.

Furthermore, recent findings suggest that there are new prognostic factors for diabetes that are not addressed in economic models. This is the case of biomarkers as C-peptide,^62,63^ the metabolic memory or legacy effect,^86,87^ the age at diabetes onset as a potential endotype,^67–69^ or the socioeconomic status of the patient’s family.

Biomarkers, such as C-peptide, might be useful as a surrogate parameter reflecting a decrease in insulin secretion.^62,63^ C-peptide levels may constitute a potential biomarker to predict the incidence of diabetic complications, and therefore, it should be considered in economic evaluations modeling the evolution of pediatric patients with diabetes. Further economic evaluations would benefit from novel research in this field, yielding to a robust establishment of the predictability of insulin decrease determined by C-peptide levels.

Regarding the legacy effect, data from the EDIC study showed that early and intensive glycemic control decreases the risk of microvascular complications, which is known as metabolic memory.^86,87^ According to the latest evidence, glycemic control in childhood/adolescence predicts metabolic control in early adulthood.^64,65^ Thus, not achieving glycemic goals during the first years after diagnosis is related to early occurrence and increased risk of developing microvascular complications, such as nephropathy, retinopathy, or cardiovascular disease.^88–91^ Given the repercussion of the legacy effect on the evolution of the pediatric patients, modeling of glycemic control at early ages should be extended, however, only one model was identified that took this aspect into consideration.^52^ In this sense, until more robust data are available, it would be beneficial for decision makers to update models to enable sensitivity analyses that incorporate the legacy effect.

The age at diabetes onset, which has been proposed as an endotype,^67^ might help to determine a particular type of diabetes. This endotype may be related to the glycemic, genetic, or metabolic disease profile and may eventually be related to a decrease in b cell count and disease prognosis.^67–69^ Nevertheless, the economic evaluations identified in the present review did not consider this factor, as it is not yet included in current clinical practice guidelines. Additionally, in the last years, the diabetes onset occurs at younger ages in the pediatric setting,^92^ which might have a remarkable impact on health outcomes, worsening the prognosis of patients with diabetes. Given that remarkable differences in results could be found depending on the endotype, decision makers would benefit from having the results of the economic evaluations based on the age at diabetes onset. This would allow them to evaluate the scenarios that best represent the context they are evaluating.

Furthermore, other factors appear to influence the achievement of glycemic goals in pediatric clinical settings and are still not included in either modeling studies or clinical practice guidelines. For instance, the socioeconomic status of parents and caregivers may be a determining factor for the achievement of glycemic goals.^70,71^ Approximately 30% to 55% of health outcomes are related to social determinants, and these are considered to be primary drivers of avoidable health inequities.^71^ For instance, lower-income regions may face challenges in accessing healthcare or adopting innovative therapies, which can significatively improve glycemic control and lead to better outcomes. Therefore, the impact of these determinants should be assessed from both clinical and economic perspectives. Decisions in this regard might vary depending on the socioeconomic context where decisions are being made.

Finally, the available evidence regarding risk equations and quality of life–related parameters represents another limitation to be considered when modeling in pediatric diabetes. There is a lack of studies including large cohorts of pediatric subjects with diabetes that were longitudinally followed up, thus, complications’ risk equations used in economic models mainly originate from studies including participants who do not fit the target population of the economic evaluations. Due to these differences, which might otherwise be expected when comparing pediatric subjects against adults, a limitation may be expected for those economic evaluations in pediatric diabetes setting. Children are vulnerable to hypoglycemia or hyperglycemia^11,12^; thus, modeling risk equations in these populations based on adults’ data would lead to inaccurate clinical and economic burden. In this sense, additional observational studies enrolling large pediatric populations, and being followed during long time horizons, are needed to assess the risk of acute and chronic diabetic complications in these patients.

Similarly, the sources of utility/disutility values are usually key drivers of uncertainty and produce remarkable variations on the models’ results. Thus, the availability of well-designed clinical studies estimating health utility/disutility values in target populations, aligned with the economic evaluations, is important to ensure the representativeness of the results for the setting where decisions are being made. Regarding pediatric diabetes, the population for those HRQoL studies estimating utility/disutility values, and for economic studies significantly differ in terms of age range and type of diabetes. Moreover, it could be argued that the utility/disutility values are specific for the region, as these are preference-based studies which are highly influenced by the regional social context. Thus, their use in other countries could lead to imprecision. In addition to the differences in age, country, and type of diabetes, the methods and tools used to assess HRQoL in patients with diabetes were remarkably heterogeneous. It is important to mention that utility values usually range from 0 to 1; thus, relatively small variations in these values (eg, 0.005) could produce remarkable differences in the results. For all these reasons, it is essential to expand the utilization of validated tools for measuring HRQoL in the target population, pediatric subjects with T1D and T2D, considering the country of origin.

Beyond the aspects evaluated in the present study, other parameters should be considered in economic evaluations focused on pediatric diabetes patients, such as the burden on caregivers. Additionally, there are broader challenges that occur universally across all models (not only in pediatric diabetes), such as advisory boards of experts, which are often used to validate assumptions and model parameters. It is challenging to assess how expert opinions might introduce bias into the analyses. For instance, assuming a negligible risk of DKA in children using CGM devices may lead to an underestimation of the associated risks. It would be valuable for future research to evaluate how assumptions based on clinicians’ judgment, rather than published evidence, could also influence the results of economic evaluations.

Thus, according to the present literature review, while economic models in pediatric diabetes provide valuable evidence for decision making, they often do not fully integrate the latest scientific advancements. Medicine and therapeutic approaches are constantly evolving, and technological innovations in medical devices are advancing rapidly, with short intervals between developments. These dynamics make it challenging to design economic models that remain up to date with the latest evidence, as they must balance the complexity of emerging data with practical modeling limitations.

Moreover, the lack of pediatric-specific sources might also produce an inaccurate reflection of the disease. Nonetheless, the actual effect of these limitations on the models remains unexplored, as most of the identified studies did not incorporate the aspects evaluated in this study, making it impossible to conduct a quantitative analysis that determines the real impact of these issues. Furthermore, in many cases, the challenge lies in the limited evidence currently available for the pediatric population under consideration, making it unfeasible to perform economic evaluations that include all of the aspects addressed in this study. Therefore, further research is needed not only to assess the impact of including these factors but also to allow for the effective incorporation of the evaluated aspects into modeling.

### Limitations

Although this study included a wide variety of information on economic modeling in pediatric diabetes, there are some limitations that must be addressed. First, the literature review was conducted in a single database (PubMed). Therefore, some relevant studies might not have been identified. Although a selection bias could be expected, the conclusions of the study are not expected to change remarkably, as many models used in pediatric diabetes are predesigned (eg, CORE Diabetes Model). In addition, to address this limitation, the previously published SLR reports were reviewed in depth to extract the 10 additional economic evaluations which were not found in PubMed. Evidence published in scientific conferences or other non-peer-reviewed sources (“gray literature”) was not included in this study. This decision was based on the fact that most gray literature does not provide sufficient details about model design and parameters to meet the objectives of this study.

Additionally, as some economic models or their respective Technical Reports were not available, the corresponding data were collected from reports of these studies. Consequently, a lack of completeness of the model programming might be expected in some cases.

### Strengths

This is the first study designed to assess the methodological issues of pediatric diabetes models specifically. Given that pediatric patients present remarkable clinical differences compared with an adult setting, the pediatric-specific design of the search strategy could be considered the main strength of the review. Another strength of the study is that it provides not only a detailed insight into the modeling approaches available for pediatric diabetes but also an exhaustive summary of the latest scientific findings in the field of study based on the clinical practice guidelines and the opinion of pediatric endocrinologists with extensive clinical and research experience in pediatric diabetes.

## CONCLUSION

Modeling long-term health and cost outcomes interventions is useful in environments of cost-containment to inform healthcare decision making. Effective modeling implies the use of data sources and parameters in alignment with the target population, eg, risk equations and utility values. However, due to the lack of evidence specific to pediatric diabetes, economic models in this field present certain limitations. In addition, current models do not incorporate novel CGM metrics (TIR, TAR, and TBR) that, together with HbA1c and hypoglycemia events, could result in a more complete picture of glycemic profiles. Moreover, aspects such as the C-peptide, the legacy effect, the age at diabetes onset, or socioeconomic status should also be considered in terms of disease prognosis. All these parameters could improve the accuracy of long-term outcome predictions, thereby enhancing cost-effectiveness analysis.

The inclusion of these latest scientific findings in economic models for pediatric diabetes could provide a more faithful reflection of the disease. However, further clinical research focusing on the assessment of all these gaps should be conducted to enhance evidence-based health economic modeling that best represents reality. Until then, with regard to pediatric diabetes, decision makers should interpret results obtained through economic models with caution and rely on additional available evidence to assess the usefulness of new diabetes therapies.

## Supplementary Material

Online Supplementary Material
