# Supplementary material for: Are Trends in Economic Modeling of Pediatric Diabetes Mellitus up to Date with the Clinical Practice Guidelines and the Latest Scientific Findings?
Source: J Health Econ Outcomes Res. 2025 Feb 3;12(1):30–50. doi: 10.36469/001c.127920 (PMC11797704; doi:10.36469/001c.127920)
Supplement: Online Supplementary Material [file jheor_2025_12_1_127920_264469.pdf]

Journal of Health Economics and Outcomes Research

Online Supplementary Material

Are Trends in Economic Modeling of Pediatric Diabetes Mellitus up to Date With the Clinical Practice Guidelines and the Latest Scientific Findings? *JHEOR*. 2025;12(1):30-50. doi:10.36469/jheor.2025.127920

Table S1: Search Strategy Designed to Find Studies Consisting of Economic Evaluations of Interventions in Pediatric Diabetes

Table S2: Search Strategy for Previous SLR and/or MA Including Economic Evaluations of Interventions in Pediatric Diabetes

Table S3: Inclusion and Exclusion Criteria for the Literature Review

Table S4: Quality Assessment of the Studies by the CHEERS 2022 Checklist

Table S5: Clinical Practice Guidelines Consulted (References)

Table S6: Sources of Utility/Disutility Values (References)

Figure S1: PRISMA Flowchart for SLR and MA Identification, Screening, and Inclusion

This supplementary material has been provided by the authors to give readers additional information about their work.

This is an open-access article distributed under the terms of the Creative Commons Attribution 4.0 International License (CCBY-4.0). View this license's legal deed at http://creativecommons.org/licenses/by/4.0 and legal code at http://creativecommons.org/licenses/by/4.0/legalcode for more information.

Table S1. Search Strategy Designed to Find Studies Consisting of Economic Evaluations of Interventions in Pediatric Diabetes

**Terms**		**Search Strategy**	**Results**
**Pathology**			
#1	Diabetes	"Diabetes Mellitus"[MeSH Terms] OR "diabet*"[Title/Abstract]	823 629
Economic evaluation			
#2	Cost-effectiveness analysis	"cost effectiveness analysis"[MeSH Terms] OR ("Cost-Effectiveness"[TIAB]) OR ("Cost Effectiveness"[TIAB]) OR ("Cost-Effective"[TIAB]) OR ("Cost Effective"[TIAB])	168 614
#3	Cost-utility analysis	("cost benefit analysis"[MeSH Terms]) OR ("Cost-Utility"[TIAB]) OR ("Cost Utility"[TIAB]) OR ("Cost-Benefit"[TIAB]) OR ("Cost Benefit"[TIAB])	101 254
#4	Economic model	("Economic"[TIAB]) AND ("Model*"[TIAB])	65 629
#5	#2 OR #3 OR #4	(("cost effectiveness analysis"[MeSH Terms] OR ("Cost-Effectiveness"[TIAB]) OR ("Cost Effectiveness"[TIAB]) OR ("Cost-Effective"[TIAB]) OR ("Cost Effective"[TIAB])) OR (("cost benefit analysis"[MeSH Terms]) OR ("Cost-Utility"[TIAB]) OR ("Cost Utility"[TIAB]) OR ("Cost-Benefit"[TIAB]) OR ("Cost Benefit"[TIAB]))) OR (("Economic"[TIAB]) AND ("Model*"[TIAB]))	272 604
**Economic evaluation in diabetes**			
#6	#1 AND #5	("Diabetes Mellitus"[MeSH Terms] OR "diabet*"[Title/Abstract]) AND ((("cost effectiveness analysis"[MeSH Terms] OR ("Cost-Effectiveness"[TIAB]) OR ("Cost Effectiveness"[TIAB]) OR ("Cost-Effective"[TIAB]) OR ("Cost Effective"[TIAB])) OR (("cost benefit analysis"[MeSH Terms]) OR ("Cost-Utility"[TIAB]) OR ("Cost Utility"[TIAB]) OR ("Cost-Benefit"[TIAB]) OR ("Cost Benefit"[TIAB]))) OR (("Economic"[TIAB]) AND ("Model*"[TIAB])))	9 588
**Language**			
#7	English/Spanish	"English"[Lang] OR "Spanish"[Lang]	30 915 455
#8	#6 AND #7	(("Diabetes Mellitus"[MeSH Terms] OR "diabet*"[Title/Abstract]) AND ((("cost effectiveness analysis"[MeSH Terms] OR ("Cost-Effectiveness"[TIAB]) OR ("Cost Effectiveness"[TIAB]) OR ("Cost-Effective"[TIAB]) OR ("Cost Effective"[TIAB])) OR (("cost benefit analysis"[MeSH Terms]) OR ("Cost-Utility"[TIAB]) OR ("Cost Utility"[TIAB]) OR ("Cost-Benefit"[TIAB]) OR ("Cost Benefit"[TIAB]))) OR (("Economic"[TIAB]) AND ("Model*"[TIAB])))) AND ("English"[Lang] OR "Spanish"[Lang])	9261
**Type of study**			
#9	Type of study filter	"review"[Filter] OR "systematicreviews"[Filter] OR "meta-analysis"[Filter] OR "randomized controlled trial, veterinary"[Filter] OR "randomized controlled trial"[Filter] OR "qualitative research/broad"[Filter] OR "qualitative research/narrow"[Filter] OR "clinical trial - phase i"[Filter] OR "clinical trial - phase ii"[Filter] OR "clinical trial - phase iii"[Filter] OR "clinical trial - phase iv"[Filter] OR "clinical trial protocol"[Filter] OR "clinical trial"[Filter] OR "observational study"[Filter] OR "address"[Filter] OR "autobiography"[Filter] OR "bibliography"[Filter] OR "biography"[Filter] OR "case reports"[Filter] OR "clinical conference"[Filter] OR "clinical description"[Filter] OR "clinical prediction guides/broad"[Filter] OR "clinical prediction guides/narrow"[Filter] OR "clinical study"[Filter] OR "comment"[Filter] OR "consensus development conference - nih"[Filter] OR "consensus development conference"[Filter] OR "dictionary"[Filter] OR "editorial"[Filter] OR "festschrift"[Filter] OR "guideline"[Filter] OR "historical article"[Filter] OR "interactive tutorial"[Filter] OR "interview"[Filter] OR "introductory journal article"[Filter] OR "lecture"[Filter] OR "legal case"[Filter] OR "legislation"[Filter] OR "letter"[Filter] OR "news"[Filter] OR "patient education handout"[Filter] OR "periodical index"[Filter] OR "personal narrative"[Filter] OR "practice guideline"[Filter] OR "published erratum"[Filter] OR "retracted publication"[Filter] OR "retraction of publication"[Filter] OR "scientific integrity review"[Filter] OR "twin study"[Filter] OR "video-audio media"[Filter] OR "webcast"[Filter]	17 555 491

Table S1. Search Strategy Designed to Find Studies Consisting of Economic Evaluations of Interventions in Pediatric Diabetes

**Terms**		**Search Strategy**	**Results**
#10	#8 NOT #9	(((“Diabetes Mellitus”[MeSH Terms] OR “diabet*”[Title/Abstract]) AND (((“cost effectiveness analysis”[MeSH Terms] OR (“Cost-Effectiveness”[TIAB]) OR (“Cost Effectiveness”[TIAB]) OR (“Cost-Effective”[TIAB]) OR (“Cost Effective”[TIAB])) OR ((“cost benefit analysis”[MeSH Terms]) OR (“Cost-Utility”[TIAB]) OR (“Cost Utility”[TIAB]) OR (“Cost-Benefit”[TIAB]) OR (“Cost Benefit”[TIAB]))) OR ((“Economic”[TIAB]) AND (“Model*”[TIAB])))) AND (“English”[Lang] OR “Spanish”[Lang])) NOT (“review”[Filter] OR “systematicreviews”[Filter] OR “meta-analysis”[Filter] OR “randomized controlled trial, veterinary”[Filter] OR “randomized controlled trial”[Filter] OR “qualitative research/broad”[Filter] OR “qualitative research/narrow”[Filter] OR “clinical trial - phase i”[Filter] OR “clinical trial - phase ii”[Filter] OR “clinical trial - phase iii”[Filter] OR “clinical trial - phase iv”[Filter] OR “clinical trial protocol”[Filter] OR “clinical trial”[Filter] OR “observational study”[Filter] OR “address”[Filter] OR “autobiography”[Filter] OR “bibliography”[Filter] OR “biography”[Filter] OR “case reports”[Filter] OR “clinical conference”[Filter] OR “clinical description”[Filter] OR “clinical prediction guides/broad”[Filter] OR “clinical prediction guides/narrow”[Filter] OR “clinical study”[Filter] OR “comment”[Filter] OR “consensus development conference - nih”[Filter] OR “consensus development conference”[Filter] OR “dictionary”[Filter] OR “editorial”[Filter] OR “festschrift”[Filter] OR “guideline”[Filter] OR “historical article”[Filter] OR “interactive tutorial”[Filter] OR “interview”[Filter] OR “introductory journal article”[Filter] OR “lecture”[Filter] OR “legal case”[Filter] OR “legislation”[Filter] OR “letter”[Filter] OR “news”[Filter] OR “patient education handout”[Filter] OR “periodical index”[Filter] OR “personal narrative”[Filter] OR “practice guideline”[Filter] OR “published erratum”[Filter] OR “retracted publication”[Filter] OR “retraction of publication”[Filter] OR “scientific integrity review”[Filter] OR “twin study”[Filter] OR “video-audio media”[Filter] OR “webcast”[Filter])	2367
**Population**			
#11	Pediatric age (0-25 years-old)	“pediatr*”[TIAB] OR “paediatr*”[TIAB] “pediatrics”[MeSH Terms] OR “adolescent”[MeSH Terms] OR “infant”[MeSH Terms] OR “child”[MeSH Terms] OR “adolescent”[MeSH Terms] OR “infant”[MeSH Terms] OR “child”[MeSH Terms:noexp] OR “child”[MeSH Terms:noexp] OR “infant”[MeSH Terms:noexp] OR “infant”[MeSH Terms:noexp] OR “young adult”[MeSH Terms:noexp] OR “infant, newborn”[MeSH Terms] OR “child, preschool”[MeSH Terms] OR “adolescent”[MeSH Terms] OR “young adult”[MeSH Terms] OR “adolescent”[TIAB] OR “youth”[TIAB] OR “youths”[TIAB] OR “youth s”[TIAB] OR “kids”[TIAB] OR “child”[MeSH Terms] OR “child”[TIAB] OR “children”[TIAB] OR “child s”[TIAB] OR “children s”[TIAB] OR “childrens”[TIAB] OR “childs”[TIAB] OR “Young adult”[TIAB] OR “Young adults”[TIAB]	4773328
Final search			
#12	#10 AND #11	((((“Diabetes Mellitus”[MeSH Terms] OR “diabet*”[Title/Abstract]) AND (((“cost effectiveness analysis”[MeSH Terms] OR (“Cost-Effectiveness”[TIAB]) OR (“Cost Effectiveness”[TIAB]) OR (“Cost-Effective”[TIAB]) OR (“Cost Effective”[TIAB])) OR ((“cost benefit analysis”[MeSH Terms]) OR (“Cost-Utility”[TIAB]) OR (“Cost Utility”[TIAB]) OR (“Cost-Benefit”[TIAB]) OR (“Cost Benefit”[TIAB]))) OR ((“Economic”[TIAB]) AND (“Model*”[TIAB])))) AND (“English”[Lang] OR “Spanish”[Lang])) NOT (“review”[Filter] OR “systematicreviews”[Filter] OR “meta-analysis”[Filter] OR “randomized controlled trial, veterinary”[Filter] OR “randomized controlled trial”[Filter] OR “qualitative research/broad”[Filter] OR “qualitative research/narrow”[Filter] OR “clinical trial - phase i”[Filter] OR “clinical trial - phase ii”[Filter] OR “clinical trial - phase iii”[Filter] OR “clinical trial - phase iv”[Filter] OR “clinical trial protocol”[Filter] OR “clinical trial”[Filter] OR “observational study”[Filter] OR “address”[Filter] OR “autobiography”[Filter] OR “bibliography”[Filter] OR “biography”[Filter] OR “case reports”[Filter] OR “clinical conference”[Filter] OR “clinical description”[Filter] OR “clinical prediction guides/broad”[Filter] OR “clinical prediction guides/narrow”[Filter] OR “clinical study”[Filter] OR “comment”[Filter] OR “consensus development conference - nih”[Filter] OR “consensus development conference”[Filter] OR “dictionary”[Filter] OR “editorial”[Filter] OR “festschrift”[Filter] OR “guideline”[Filter] OR “historical article”[Filter] OR “interactive tutorial”[Filter] OR “interview”[Filter] OR “introductory journal article”[Filter] OR “lecture”[Filter] OR “legal case”[Filter] OR “legislation”[Filter] OR “letter”[Filter] OR “news”[Filter] OR “patient education handout”[Filter] OR “periodical index”[Filter] OR “personal narrative”[Filter] OR “practice guideline”[Filter] OR “published erratum”[Filter] OR “retracted publication”[Filter] OR “retraction of publication”[Filter] OR	304

Table S1. Search Strategy Designed to Find Studies Consisting of Economic Evaluations of Interventions in Pediatric Diabetes

Terms		Search Strategy	Results
#12	#10 AND #11	"scientific integrity review"[Filter] OR "twin study"[Filter] OR "video-audio media"[Filter] OR "webcast"[Filter])) AND ("pediatr*"[TIAB] OR "paediatr*"[TIAB] "pediatrics"[MeSH Terms] OR "adolescent"[MeSH Terms] OR "infant"[MeSH Terms] OR "child"[MeSH Terms] OR "adolescent"[MeSH Terms] OR "infant"[MeSH Terms] OR "child"[MeSH Terms:noexp] OR "child"[MeSH Terms:noexp] OR "infant"[MeSH Terms:noexp] OR "infant"[MeSH Terms:noexp] OR "young adult"[MeSH Terms:noexp] OR "infant, newborn"[MeSH Terms] OR "child, preschool"[MeSH Terms] OR "adolescent"[MeSH Terms] OR "young adult"[MeSH Terms] OR "adolescent"[TIAB] OR "youth"[TIAB] OR "youths"[TIAB] OR "youth s"[TIAB] OR "kids"[TIAB] OR "child"[MeSH Terms] OR "child"[TIAB] OR "children"[TIAB] OR "child s"[TIAB] OR "children s"[TIAB] OR "childrens"[TIAB] OR "childs"[TIAB] OR "Young adult"[TIAB] OR "Young adults"[TIAB])	

Table S2. Search Strategy for Previous SLR and/or MA Including Economic Evaluations of Interventions in Pediatric Diabetes

Terms		Search Strategy	Results
**Pathology**			
#1’	Diabetes	“Diabetes Mellitus”[MeSH Terms] OR “diabet*”[Title/Abstract]	823 629
**Economic evaluation**			
#2′	Cost-effectiveness analysis	“cost effectiveness analysis”[MeSH Terms] OR (“Cost-Effectiveness”[TIAB]) OR (“Cost Effectiveness”[TIAB]) OR (“Cost-Effective”[TIAB]) OR (“Cost Effective”[TIAB])	168 614
#3′	Cost-utility analysis	(“cost benefit analysis”[MeSH Terms]) OR (“Cost-Utility”[TIAB]) OR (“Cost Utility”[TIAB]) OR (“Cost-Benefit”[TIAB]) OR (“Cost Benefit”[TIAB])	101 254
#4′	Economic model	(“Economic”[TIAB]) AND (“Model*”[TIAB])	65 629
#5′	#2′ OR #3′ OR #4′	((“cost effectiveness analysis”[MeSH Terms] OR (“Cost-Effectiveness”[TIAB]) OR (“Cost Effectiveness”[TIAB]) OR (“Cost-Effective”[TIAB]) OR (“Cost Effective”[TIAB])) OR ((“cost benefit analysis”[MeSH Terms]) OR (“Cost-Utility”[TIAB]) OR (“Cost Utility”[TIAB]) OR (“Cost-Benefit”[TIAB]) OR (“Cost Benefit”[TIAB]))) OR ((“Economic”[TIAB]) AND (“Model*”[TIAB]))	272 604
**Economic evaluation in diabetes**			
#6′	#1′ AND #5′	(“Diabetes Mellitus”[MeSH Terms] OR “diabet*”[Title/Abstract]) AND (((“cost effectiveness analysis”[MeSH Terms] OR (“Cost-Effectiveness”[TIAB]) OR (“Cost Effectiveness”[TIAB]) OR (“Cost-Effective”[TIAB]) OR (“Cost Effective”[TIAB])) OR ((“cost benefit analysis”[MeSH Terms]) OR (“Cost-Utility”[TIAB]) OR (“Cost Utility”[TIAB]) OR (“Cost-Benefit”[TIAB]) OR (“Cost Benefit”[TIAB]))) OR ((“Economic”[TIAB]) AND (“Model*”[TIAB])))	9 588
**Language**			
#7′	English / Spanish	“English”[Lang] OR “Spanish”[Lang]	30 915 455
#8′	#6′ AND #7′	((“Diabetes Mellitus”[MeSH Terms] OR “diabet*”[Title/Abstract]) AND (((“cost effectiveness analysis”[MeSH Terms] OR (“Cost-Effectiveness”[TIAB]) OR (“Cost Effectiveness”[TIAB]) OR (“Cost-Effective”[TIAB]) OR (“Cost Effective”[TIAB])) OR ((“cost benefit analysis”[MeSH Terms]) OR (“Cost-Utility”[TIAB]) OR (“Cost Utility”[TIAB]) OR (“Cost-Benefit”[TIAB]) OR (“Cost Benefit”[TIAB]))) OR ((“Economic”[TIAB]) AND (“Model*”[TIAB])))) AND (“English”[Lang] OR “Spanish”[Lang])	9 261
**Type of study**			
#9′	Type of study filter	“systematicreviews”[Filter] OR “meta-analysis”[Filter]	340 453
#10′	#8′ AND #10′	(((“Diabetes Mellitus”[MeSH Terms] OR “diabet*”[Title/Abstract]) AND (((“cost effectiveness analysis”[MeSH Terms] OR (“Cost-Effectiveness”[TIAB]) OR (“Cost Effectiveness”[TIAB]) OR (“Cost-Effective”[TIAB]) OR (“Cost Effective”[TIAB])) OR ((“cost benefit analysis”[MeSH Terms]) OR (“Cost-Utility”[TIAB]) OR (“Cost Utility”[TIAB]) OR (“Cost-Benefit”[TIAB]) OR (“Cost Benefit”[TIAB]))) OR ((“Economic”[TIAB]) AND (“Model*”[TIAB])))) AND (“English”[Lang] OR “Spanish”[Lang])) AND (“systematicreviews”[Filter] OR “meta-analysis”[Filter])	582
**Population**			
#11′	Pediatric age (0-25 years-old)	“pediatr*”[TIAB] OR “paediatr*”[TIAB] “pediatrics”[MeSH Terms] OR “adolescent”[MeSH Terms] OR “infant”[MeSH Terms] OR “child”[MeSH Terms] OR “adolescent”[MeSH Terms] OR “infant”[MeSH Terms] OR “child”[MeSH Terms:noexp] OR “child”[MeSH Terms:noexp] OR “infant”[MeSH Terms:noexp] OR “infant”[MeSH Terms:noexp] OR “young adult”[MeSH Terms:noexp] OR “infant, newborn”[MeSH Terms] OR “child, preschool”[MeSH Terms] OR “adolescent”[MeSH Terms] OR “young adult”[MeSH Terms] OR “adolescent”[TIAB] OR “youth”[TIAB] OR “youths”[TIAB] OR “youth s”[TIAB] OR “kids”[TIAB] OR “child”[MeSH Terms] OR “child”[TIAB] OR “children”[TIAB] OR “child s”[TIAB] OR “children s”[TIAB] OR “childrens”[TIAB] OR “childs”[TIAB] OR “Young adult”[TIAB] OR “Young adults”[TIAB]	4 773 328
**Final search**			
#12′	#10′ AND #11′	((((“Diabetes Mellitus”[MeSH Terms] OR “diabet*”[Title/Abstract]) AND (((“cost effectiveness analysis”[MeSH Terms] OR (“Cost-Effectiveness”[TIAB]) OR (“Cost Effectiveness”[TIAB]) OR (“Cost-Effective”[TIAB]) OR (“Cost Effective”[TIAB])) OR ((“cost benefit analysis”[MeSH Terms]) OR (“Cost-Utility”[TIAB]) OR (“Cost Utility”[TIAB]) OR (“Cost-Benefit”[TIAB]) OR (“Cost Benefit”[TIAB]))) OR ((“Economic”[TIAB]) AND (“Model*”[TIAB])))) AND (“English”[Lang] OR “Spanish”[Lang])) AND (“systematicreviews”[Filter] OR “meta-analysis”[Filter])) AND (“pediatr*”[TIAB] OR	

Table S2. Search Strategy for Previous SLR and/or MA Including Economic Evaluations of Interventions in Pediatric Diabetes

Terms		Search Strategy	Results
#12′	#10′AND #11′	"paediatr*"[TIAB] "pediatrics"[MeSH Terms] OR "adolescent"[MeSH Terms] OR "infant"[MeSH Terms] OR "child"[MeSH Terms] OR "adolescent"[MeSH Terms] OR "infant"[MeSH Terms] OR "child"[MeSH Terms:noexp] OR "child"[MeSH Terms:noexp] OR "infant"[MeSH Terms:noexp] OR "infant"[MeSH Terms:noexp] OR "young adult"[MeSH Terms:noexp] OR "infant, newborn"[MeSH Terms] OR "child, preschool"[MeSH Terms] OR "adolescent"[MeSH Terms] OR "young adult"[MeSH Terms] OR "adolescent"[TIAB] OR "youth"[TIAB] OR "youths"[TIAB] OR "youth s"[TIAB] OR "kids"[TIAB] OR "child"[MeSH Terms] OR "child"[TIAB] OR "children"[TIAB] OR "child s"[TIAB] OR "children s"[TIAB] OR "childrens"[TIAB] OR "childs"[TIAB] OR "Young adult"[TIAB] OR "Young adults"[TIAB])	70

Abbreviations: MA, meta-analysis; SLR, systematic literature review.

Table S3. Inclusion and Exclusion Criteria for the Literature Review

Inclusion Criteria	Exclusion Criteria
Studies that included patients with DM<br>Studies that included pediatric patients (<25 years old)<br>Partial or completed economic evaluation of health interventions<br>Partial or completed economic evaluations conducted by designing economic models<br>Studies published in English or Spanish<br>Studies published in any date	Studies about different pathologies than DM<br>Studies that included patients >25 years old<br>Studies different than partial or complete economic evaluations, eg, clinical trials, observational studies, qualitative research, etc<br>Studies published in different languages than English or Spanish

Abbreviation: DM, diabetes mellitus.

Table S4. Quality Assessment of the Studies by the CHEERS 2022 Checklist

References	1	2	3	4	5	6	7	8	9	10	11	12	13	14	15	16	17	18	19	20	21	22	23	24	25	26	27	28
Javitt (1990)	✓	✓	✓	X	✓	✓	✓	✓	✓	✓	✓	✓	✓	✓	✓	✓	✓	✓	-	✓	✓	✓	✓	✓	-	✓	X	✓
Palmer (2000)	✓	✓	✓	X	✓	✓	✓	✓	✓	✓	✓	✓	✓	✓	✓	✓	✓	✓	-	✓	-	✓	✓	✓	-	✓	✓	✓
Roze (2005)	✓	✓	✓	X	✓	✓	✓	✓	✓	✓	✓	✓	✓	✓	✓	✓	✓	✓	-	✓	-	✓	✓	✓	-	✓	✓	✓
Cohen (2007)	✓	✓	✓	X	✓	✓	✓	✓	✓	✓	✓	✓	✓	✓	✓	✓	✓	✓	-	✓	-	✓	✓	✓	-	✓	✓	✓
Dall (2009)	✓	✓	✓	X	✓	✓	✓	✓	X	✓	-	-	-	✓	✓	✓	✓	X	-	X	-	✓	✓	X	-	✓	✓	✓
Gschwend (2009)	✓	✓	✓	X	✓	✓	✓	✓	✓	✓	X	X	X	X	X	✓	✓	✓	-	✓	-	✓	✓	✓	-	✓	✓	✓
Beckwith (2011)	✓	✓	✓	X	✓	✓	✓	✓	✓	✓	✓	✓	✓	✓	✓	✓	✓	✓	-	✓	-	✓	✓	✓	-	✓	X	✓
Pfhol (2012)	✓	✓	✓	X	✓	✓	✓	✓	✓	✓	✓	✓	✓	✓	✓	✓	✓	✓	-	✓	-	✓	✓	✓	-	✓	✓	✓
Gómez (2016)	X	✓	✓	X	✓	✓	✓	✓	✓	✓	✓	✓	✓	X	✓	✓	✓	✓	-	✓	-	✓	✓	✓	-	✓	✓	✓
Sussman (2016)	✓	✓	✓	X	✓	✓	✓	✓	✓	✓	✓	✓	✓	✓	✓	✓	✓	✓	-	✓	-	✓	✓	✓	-	✓	✓	✓
Dawoud (2017)	✓	✓	✓	X	✓	✓	✓	✓	✓	✓	✓	✓	✓	✓	✓	✓	✓	✓	-	✓	-	✓	✓	✓	-	✓	✓	✓
Roze (2017)	✓	✓	✓	X	✓	✓	✓	✓	✓	✓	✓	✓	✓	✓	✓	✓	✓	✓	-	✓	-	✓	✓	✓	-	✓	✓	✓
Thomas (2017)	✓	✓	✓	X	✓	✓	✓	✓	✓	✓	✓	✓	✓	✓	✓	✓	✓	✓	-	✓	-	✓	✓	✓	-	✓	✓	✓
GoodSmith (2019)	✓	✓	✓	X	✓	✓	✓	✓	✓	✓	✓	✓	✓	✓	✓	✓	✓	✓	-	✓	-	✓	✓	✓	-	✓	✓	✓
Roze (2019)	✓	✓	✓	X	✓	✓	✓	✓	✓	✓	✓	✓	✓	✓	✓	✓	✓	✓	-	✓	-	✓	✓	✓	-	✓	✓	✓
McQueen (2020)	✓	✓	✓	X	✓	✓	✓	✓	✓	✓	✓	✓	✓	✓	X	✓	✓	✓	-	✓	-	✓	✓	✓	-	✓	✓	✓
Roze (2021)	✓	✓	✓	X	✓	✓	✓	✓	✓	✓	✓	✓	✓	✓	✓	✓	✓	✓	-	✓	-	✓	✓	✓	-	✓	✓	✓
Pease (2022)	✓	✓	✓	X	✓	✓	✓	✓	✓	✓	✓	✓	✓	✓	✓	✓	✓	✓	-	✓	-	✓	✓	✓	-	✓	✓	✓
Zhang (2022)	✓	✓	✓	X	✓	✓	✓	✓	✓	✓	✓	✓	✓	✓	X	✓	✓	✓	-	✓	-	✓	✓	✓	-	✓	✓	✓

Abbreviation: CHEERS, Consolidated Health Economic Evaluation Reporting Standards.

Table S5. Clinical Practice Guidelines Consulted (References)

Country	Reference
Australia	Peña AS, Curran JA, Fuery M, et al. Screening, assessment and management of type 2 diabetes mellitus in children and adolescents: Australasian Paediatric Endocrine Group guidelines. *Med J Aust*. 2020;213:30-43.
China	Diabetes Association Of The Republic Of China (Taiwan). Executive summary of the DAROC clinical practice guidelines for diabetes care- 2018. *J Formos Med Assoc*. 2020;119:577-586.
Korea	Ko S-H. 2021 Clinical Practice Guidelines for Diabetes Mellitus in Korea. *J Korean Diabetes*. 2021;22:244-249.
Canada	Wherrett DK, Ho J, Huot C, et al. Type 1 diabetes in children and adolescents. *Can J Diabetes*. 2018;42:S234-S246.
United States	ElSayed NA, Aleppo G, Aroda VR, et al. Summary of Revisions: Standards of Care in Diabetes—2023. *Diabetes Care*. 2023;46:S5-S9.
	ElSayed NA, Aleppo G, Aroda VR, et al. Introduction and Methodology: Standards of Care in Diabetes—2023. *Diabetes Care*. 2023;46:S1-S4.
	ElSayed NA, Aleppo G, Aroda VR, et al. 1. Improving Care and Promoting Health in Populations: Standards of Care in Diabetes—2023. *Diabetes Care*. 2023;46:S10-S18.
	ElSayed NA, Aleppo G, Aroda VR, et al. 2. Classification and Diagnosis of Diabetes: Standards of Care in Diabetes—2023. *Diabetes Care*. 2023;46:S19-S40.
	ElSayed NA, Aleppo G, Aroda VR, et al. 3. Prevention or Delay of Type 2 Diabetes and Associated Comorbidities: Standards of Care in Diabetes—2023. *Diabetes Care*. 2023;46:S41-S48.
	ElSayed NA, Aleppo G, Aroda VR, et al. 4. Comprehensive Medical Evaluation and Assessment of Comorbidities: Standards of Care in Diabetes—2023. *Diabetes Care*. 2023;46:S49-S67.
	ElSayed NA, Aleppo G, Aroda VR, et al. 5. Facilitating Positive Health Behaviors and Well-being to Improve Health Outcomes: Standards of Care in Diabetes—2023. *Diabetes Care*. 2023;46:S68-S96.
	ElSayed NA, Aleppo G, Aroda VR, et al. 6. Glycemic Targets: Standards of Care in Diabetes—2023. *Diabetes Care*. 2023;46:S97-S110.
	ElSayed NA, Aleppo G, Aroda VR, et al. 7. Diabetes Technology: Standards of Care in Diabetes — 2023. *Diabetes Care*. 2023;46:S111-S127.
	ElSayed NA, Aleppo G, Aroda VR, et al. 8. Obesity and Weight Management for the Prevention and Treatment of Type 2 Diabetes: Standards of Care in Diabetes—2023. *Diabetes Care*. 2023;46:S128-S139.
	ElSayed NA, Aleppo G, Aroda VR, et al. 9. Pharmacologic Approaches to Glycemic Treatment: Standards of Care in Diabetes-2023. *Diabetes Care*. 2023;46:S140-S157.
	ElSayed NA, Aleppo G, Aroda VR, et al. 10. Cardiovascular Disease and Risk Management: Standards of Care in Diabetes—2023. *Diabetes Care*. 2023;46:S158-S190.
	ElSayed NA, Aleppo G, Aroda VR, et al. 11. Chronic Kidney Disease and Risk Management: Standards of Care in Diabetes—2023. *Diabetes Care*. 2023;46:S191-S202.
	ElSayed NA, Aleppo G, Aroda VR, et al. 12. Retinopathy, Neuropathy, and Foot Care: Standards of Care in Diabetes—2023. *Diabetes Care*. 2023;46:S203-S215.
	ElSayed NA, Aleppo G, Aroda VR, et al. 13. Older Adults: Standards of Care in Diabetes—2023. *Diabetes Care*. 2023;46:S216-S229.
	ElSayed NA, Aleppo G, Aroda VR, et al. 14. Children and Adolescents: Standards of Care in Diabetes—2023. *Diabetes Care*. 2023;46:S230-S253.
	ElSayed NA, Aleppo G, Aroda VR, et al. 15. Management of Diabetes in Pregnancy: Standards of Care in Diabetes—2023. *Diabetes Care*. 2023;46:S254-S266.
	American Diabetes Association. 16. Diabetes Advocacy: Standards of Medical Care in Diabetes-2021. *Diabetes Care*. 2021;44:S221-S222.
	ElSayed NA, Aleppo G, Aroda VR, et al. 17. Diabetes Advocacy: Standards of Care in Diabetes—2023. *Diabetes Care*. 2023;46:S279-S280.
	Disclosures: Standards of Care in Diabetes—2023. *Diabetes Care*. S281-S284.
	Chamberlain JJ, Doyle-Delgado K, Peterson L, et al. Diabetes Technology: Review of the 2019 American Diabetes Association Standards of Medical Care in Diabetes. Ann Intern Med. 2019;171:415.
	McCall AL, Lieb DC, Gianchandani R, et al. Management of Individuals With Diabetes at High Risk for Hypoglycemia: An Endocrine Society Clinical Practice Guideline. *J Clin Endocrinol Metab*. 2023;108:529-562.
	Copeland KC, Silverstein J, Moore KR, et al. Management of Newly Diagnosed Type 2 Diabetes Mellitus (T2DM) in Children and Adolescents. *Pediatrics*. 2013;131:364-382.

Table S5. Clinical Practice Guidelines Consulted (References)

Country	Reference
United States	Doyle-Delgado K, Chamberlain JJ, Shubrook JH, et al. Pharmacologic Approaches to Glycemic Treatment of Type 2 Diabetes: Synopsis of the 2020 American Diabetes Association's Standards of Medical Care in Diabetes Clinical Guideline. *Ann Intern Med*. 2020;173:813-821.
	Springer SC, Silverstein J, Copeland K, et al. Management of Type 2 Diabetes Mellitus in Children and Adolescents. *Pediatrics*. 2013;131:e648-e664.
	Blonde L, Umpierrez GE, Reddy SS, et al. American Association of Clinical Endocrinology Clinical Practice Guideline: Developing a Diabetes Mellitus Comprehensive Care Plan—2022 Update. *Endocr Pract*. 2022;28:923-1049.
Germany	Heinemann L, Deiss D, Siegmund T, et al. Practical Recommendations for Glucose Measurement, Glucose Monitoring and Glucose Control in Patients with Type 1 or Type 2 Diabetes in Germany. *Exp Clin Endocrinol Diabetes*. 2018;126:411-428.
Italy	d'Annunzio G, Maffeis C, Cherubini V, et al. Caring for children and adolescents with type 1 diabetes mellitus: Italian Society for Pediatric Endocrinology and Diabetology (ISPED) statements during COVID-19 pandemia. *Diabetes Res Clin Pract*. 2020;168:108372.
Spain	Arrieta F, Pedro-Botet J, Iglesias P, et al. Diabetes mellitus y riesgo cardiovascular: actualización de las recomendaciones del Grupo de Trabajo de Diabetes y Enfermedad Cardiovascular de la Sociedad Española de Diabetes (SED, 2021). *Clín Invest Arterioscler*. 2022;34:36-55.
	Enríquez Jiménez M, Navarro Antón C, Peralta Pérez G, et al. Manual de insulinización para enfermería. Madrid: IMC, International Marketing & Communication, S.A.; 2021.
	Franco JM, Girbés Borrás J, Casañ Fernández R, et al. Guía para la prescripción y visado de antidiabéticos [Internet]. Valencia: Instituto Médico Valenciano; 2019. Available from: https://www.semg.es/images/documentos/guia-visado-antidiabeticos-20190120.pdf#:~:text=El%20objetivo%20es%20establecer%20los%20requisitos%20m%C3%ADnimos%20que,tratamiento%20y%20evitar%C3%A1%20molestias%20innecesarias%20a%20nuestros%20pacientes.
	Gomez-Peralta F, Escalada San Martín FJ, Menéndez Torre E, et al. Recomendaciones de la Sociedad Española de Diabetes (SED) para el tratamiento farmacológico de la hiperglucemia en la diabetes tipo 2: Actualización 2018. *Endocrinol Diabetes Nutr*. 2018;65:611-624.
	Grupo de Gestión Sanitaria, Grupo de Educación Terapéutica, Sociedad Española de Diabetes (SED). Posicionamiento de la sociedad española de diabetes sobre la validación de las guías para la indicación, uso y autorización de dispensación de medicamentos sujetos a prescripción médica por parte de las/los enfermeras/os de hipertensión y de diabetes mellitus tipo 1 y tipo 2 por parte de la dirección general de salud pública del ministerio de sanidad. [Internet]. Madrid: SED; 2022. Available from: https://www.sediabetes.org/wp-content/uploads/BOE-A-2022-11127-POSICIONAMIENTO-SOCIEDAD-ESPANOLA-DE-DIABETES-1.pdf.
	Grupo de trabajo de la Guía de Práctica Clínica sobre Diabetes mellitus tipo 1. Guía de Práctica Clínica sobre Diabetes Mellitus Tipo 1 [Internet]. Vitoria-Gasteiz: Osteba; 2012. Available from: https://www.euskadi.eus/contenidos/informacion/osteba_publicaciones/es_osteba/adjuntos/GPC12_01c.pdf.
United Kingdom	National Institute for Health and Care Excellence (NICE). 2022 exceptional surveillance of diabetes (type 1 and type 2) in children and young people: diagnosis and management (NICE guideline NG18) [Internet]. London: NICE; 2022. Available from: www.nice.org.uk.
	National Institute for Health and Care Excellence (NICE). Diabetes (type 1 and type 2) in children and young people: diagnosis and management [Internet]. London: NICE; 2015. Available from: www.nice.org.uk/guidance/ng18.
	Beckles ZL, Edge JA, Mugglestone MA, et al. Diagnosis and management of diabetes in children and young people: summary of updated NICE guidance. *BMJ*. 2016;i139.
	Choudhary P, Campbell F, Joule N, et al. A Type 1 diabetes technology pathway: consensus statement for the use of technology in Type 1 diabetes. *Diabet Med*. 2019;36:531-538.
European Countries (Collaboration)	Buse JB, Wexler DJ, Tsapas A, et al. 2019 update to: Management of hyperglycaemia in type 2 diabetes, 2018. A consensus report by the American Diabetes Association (ADA) and the European Association for the Study of Diabetes (EASD). *Diabetologia*. 2020;63:221-228.
	Riddle MC, Cefalu WT, Evans PH, et al. Consensus report: definition and interpretation of remission in type 2 diabetes. *Diabetologia*. 2021;64:2359-2366.

Table S5. Clinical Practice Guidelines Consulted (References)

Country	Reference
European Countries (Collaboration)	ESC Guidelines on diabetes, pre-diabetes, and cardiovascular diseases developed in collaboration with the EASD. The Task Force on diabetes, pre-diabetes, and cardiovascular diseases of the European Society of Cardiology (ESC) and developed in collaboration with the European Association for the Study of Diabetes (EASD). *Rev Esp Cardiol Engl Ed*. 2014;67:136.
	Rydén L, Standl E, Bartnik M, et al. Guías de práctica clínica sobre diabetes, prediabetes y enfermedades cardiovasculares: versión resumida. *Rev Esp Cardiol*. 2007;60:525.e1-525.e64.
	Davies MJ, Aroda VR, Collins BS, et al. Management of hyperglycaemia in type 2 diabetes, 2022. A consensus report by the American Diabetes Association (ADA) and the European Association for the Study of Diabetes (EASD). *Diabetologia*. 2022;65:1925-1966.
	Holt RIG, DeVries JH, Hess-Fischl A, et al. The management of type 1 diabetes in adults. A consensus report by the American Diabetes Association (ADA) and the European Association for the Study of Diabetes (EASD). *Diabetologia*. 2021;64:2609-2652.
International Collaboration	Peters AL, Ahmann AJ, Battelino T, et al. Diabetes Technology—Continuous Subcutaneous Insulin Infusion Therapy and Continuous Glucose Monitoring in Adults: An Endocrine Society Clinical Practice Guideline. *J Clin Endocrinol Metab*. 2016;101:3922-3937.
	Moser O, Riddell MC, Eckstein ML, et al. Glucose management for exercise using continuous glucose monitoring (CGM) and intermittently scanned CGM (isCGM) systems in type 1 diabetes: position statement of the European Association for the Study of Diabetes (EASD) and of the International Society for Pediatric and Adolescent Diabetes (ISPAD) endorsed by JDRF and supported by the American Diabetes Association (ADA). *Diabetologia*. 2020;63:2501-2520.
	Chung WK, Erion K, Florez JC, et al. Precision medicine in diabetes: a Consensus Report from the American Diabetes Association (ADA) and the European Association for the Study of Diabetes (EASD). *Diabetologia*. 2020;63:1671-1693.
	Abraham MB, Karges B, Dovc K, et al. ISPAD Clinical Practice Consensus Guidelines 2022: Assessment and management of hypoglycemia in children and adolescents with diabetes. *Pediatr Diabetes*. 2022;23:1322-1340.
	Adolfsson P, Taplin CE, Zaharieva DP, et al. ISPAD Clinical Practice Consensus Guidelines 2022: Exercise in children and adolescents with diabetes. *Pediatr Diabetes*. 2022;23:1341-1372.
	Annan SF, Higgins LA, Jelleryd E, et al. ISPAD Clinical Practice Consensus Guidelines 2022: Nutritional management in children and adolescents with diabetes. *Pediatr Diabetes*. 2022;23:1297-1321.
	Besser REJ, Bell KJ, Couper JJ, et al. ISPAD Clinical Practice Consensus Guidelines 2022: Stages of type 1 diabetes in children and adolescents. *Pediatr Diabetes*. 2022;23:1175-1187.
	Bjornstad P, Dart A, Donaghue KC, et al. ISPAD Clinical Practice Consensus Guidelines 2022: Microvascular and macrovascular complications in children and adolescents with diabetes. *Pediatr Diabetes*. 2022;23:1432-1450.
	Bruggeman BS, Schatz DA. The ISPAD Clinical Practice Consensus Guidelines 2022: how far we have come and the distance still to go. *Lancet Diabetes Endocrinol.* 2023;11:304-307.
	Cengiz E, Danne T, Ahmad T, et al. ISPAD Clinical Practice Consensus Guidelines 2022: Insulin treatment in children and adolescents with diabetes. *Pediatr Diabetes*. 2022;23:1277-1296.
	Craig ME, Codner E, Mahmud FH, et al. ISPAD Clinical Practice Consensus Guidelines 2022: Editorial. *Pediatr Diabetes*. 2022;23:1157-1159.
	de Bock M, Codner E, Craig ME, et al. ISPAD Clinical Practice Consensus Guidelines 2022: Glycemic targets and glucose monitoring for children, adolescents, and young people with diabetes. *Pediatr Diabetes*. 2022;23:1270-1276.
	de Wit M, Gajewska KA, Goethals ER, et al. ISPAD Clinical Practice Consensus Guidelines 2022: Psychological care of children, adolescents and young adults with diabetes. *Pediatr Diabetes*. 2022;23:1373-1389.
	Deeb A, Babiker A, Sedaghat S, et al. ISPAD Clinical Practice Consensus Guidelines 2022: Ramadan and other religious fasting by young people with diabetes. *Pediatr Diabetes*. 2022;23:1512-1528.
	Fröhlich-Reiterer E, Elbarbary NS, Simmons K, et al. ISPAD Clinical Practice Consensus Guidelines 2022: Other complications and associated conditions in children and adolescents with type 1 diabetes. *Pediatr Diabetes*. 2022;23:1451-1467.
	Glaser N, Fritsch M, Priyambada L, et al. ISPAD clinical practice consensus guidelines 2022: Diabetic ketoacidosis and hyperglycemic hyperosmolar state. *Pediatr Diabetes*. 2022;23:835-856.
	Greeley SAW, Polak M, Njølstad PR, et al. ISPAD Clinical Practice Consensus Guidelines 2022: The diagnosis and management of monogenic diabetes in children and adolescents. *Pediatr Diabetes*. 2022;23:1188-1211.

Table S5. Clinical Practice Guidelines Consulted (References)

Country	Reference
International Collaboration	Gregory JW, Cameron FJ, Joshi K, et al. ISPAD Clinical Practice Consensus Guidelines 2022: Diabetes in adolescence. *Pediatr Diabetes*. 2022;23:857-871.
	Kapellen T, Agwu JC, Martin L, et al. ISPAD clinical practice consensus guidelines 2022: Management of children and adolescents with diabetes requiring surgery. *Pediatr Diabetes*. 2022;23:1468-1477.
	Lawrence SE, Albanese-O'Neill A, Besançon S, et al. ISPAD Clinical Practice Consensus Guidelines 2022: Management and support of children and adolescents with diabetes in school. *Pediatr Diabetes*. 2022;23:1478-1495.
	Libman I, Haynes A, Lyons S, et al. ISPAD Clinical Practice Consensus Guidelines 2022: Definition, epidemiology, and classification of diabetes in children and adolescents. *Pediatr Diabetes*. 2022;23:1160-1174.
	Limbert C, Tinti D, Malik F, et al. ISPAD Clinical Practice Consensus Guidelines 2022: The delivery of ambulatory diabetes care to children and adolescents with diabetes. *Pediatr Diabetes*. 2022;23:1243-1269.
	Lindholm Olinder A, DeAbreu M, Greene S, et al. ISPAD Clinical Practice Consensus Guidelines 2022: Diabetes education in children and adolescents. *Pediatr Diabetes*. 2022;23:1229-1242.
	Ode KL, Ballman M, Battezzati A, et al. ISPAD Clinical Practice Consensus Guidelines 2022: Management of cystic fibrosis-related diabetes in children and adolescents. *Pediatr Diabetes*. 2022;23:1212-1228.
	Shah AS, Zeitler PS, Wong J, et al. ISPAD Clinical Practice Consensus Guidelines 2022: Type 2 diabetes in children and adolescents. *Pediatr Diabetes*. 2022;23:872-902.
	Sherr JL, Schoelwer M, Dos Santos TJ, et al. ISPAD Clinical Practice Consensus Guidelines 2022: Diabetes technologies: Insulin delivery. *Pediatr Diabetes*. 2022;23:1406-1431.
	Sundberg F, deBeaufort C, Krogvold L, et al. ISPAD Clinical Practice Consensus Guidelines 2022: Managing diabetes in preschoolers. *Pediatr Diabetes*. 2022;23:1496-1511.
	Tauschmann M, Forlenza G, Hood K, et al. ISPAD Clinical Practice Consensus Guidelines 2022: Diabetes technologies: Glucose monitoring. *Pediatr Diabetes*. 2022;23:1390-1405.
	Virmani A, Brink SJ, Middlehurst A, et al. ISPAD Clinical Practice Consensus Guidelines 2022: Management of the child, adolescent, and young adult with diabetes in limited resource settings. *Pediatr Diabetes*. 2022;23:1529-1551.
	Urakami T. Treatment strategy for children and adolescents with type 2 diabetes-based on ISPAD Clinical Practice Consensus Guidelines 2022. *Clin Pediatr Endocrinol*. 2023;32:125-136.

Table S6. Sources of Utility/Disutility Values (References)

1. Beaudet A, Clegg J, Thuresson P-O, Lloyd A, McEwan P. Review of utility values for economic modeling in type 2 diabetes. *Value Health*. 2014;17:462-470.
2. Clarke P, Gray A, Holman R. Estimating utility values for health states of type 2 diabetic patients using the EQ-5D (UKPDS 62). *Med Decis Mak*. 2002;22:340-9.
3. Currie CJ, Morgan CL, Poole CD, Sharplin P, Lammert M, McEwan P. Multivariate models of health-related utility and the fear of hypoglycaemia in people with diabetes. *Curr Med Res Opin*. 2006;22:1523-1534.
4. Tengs TO, Wallace A. One thousand health-related quality-of-life estimates. *Med Care*. 2000;38:583-637.
5. Nørgaard K, Scaramuzza A, Bratina N, Lalić NM, Jarosz-Chobot P, Kocsis G, et al. Routine sensor-augmented pump therapy in type 1 diabetes: the INTERPRET study. *Diabetes Technol Ther*. 2013;15:273-80.
6. Australian Institute of Health and Welfare (AIHW). The burden of disease and injury in Ausralia. Australian Fovernment. Canberra; 2003.
7. Carrington AL, Mawdsley SK, Morley M, Kincey J, Boulton AJ. Psychological status of diabetic people with or without lower limb disability. *Diabetes Res Clin Pract*. 1996;32:19-25.
8. Harris S, Mamdani M, Galbo-Jørgensen CB, Bøgelund M, Gundgaard J, Groleau D. The effect of hypoglycemia on health-related quality of life: Canadian results from a multinational time trade-off survey. *Can J Diabetes*. 2014;38:45-52.
9. McBride M, Eggleston AS, Jones T, Ly T. Health-Related Quality of Life in Patients with Type 1 Diabetes and Impaired Hypoglycaemia Awareness: The Role of Sensor-Augmented Insulin Pump Therapy with Automated Insulin Suspension. *Value Health*. 2013;16:A448.
10. Nørgaard K, Scaramuzza A, Bratina N, Lalić NM, Jarosz-Chobot P, Kocsis G, et al. Sensor-augmented pump therapy in real-life: patients reported outcomes results of the INTERPRET observational study. Abstract. Berlin: EASD; 2012. p. 1058.
11. Palmer AJ, Roze S, Valentine WJ, Minshall ME, Foos V, Lurati FM, et al. The CORE Diabetes Model: Projecting Long-term Clinical Outcomes, Costs and Costeffectiveness of Interventions in Diabetes Mellitus (Types 1 and 2) to Support Clinical and Reimbursement Decision-making. *Curr Med Res Opin*. 2004;20:S5-26.
12. Alva M, Gray A, Mihaylova B, Clarke P. The effect of diabetes complications on health-related quality of life: the importance of longitudinal data to address patient heterogeneity. *Health Econ*. 2013;10.
13. Bagust A, Beale S.Modelling EuroQol healthrelated utility values for diabetic complications from CODE-2 data. *Health Econ*. 2005;14:217-230.
14. Begg S, Vos T, Barker B, Stevenson C, Stanley L, Lopez AD. The burden of disease and injury in Australia 2003. PHE 82. 2007. Canberra: AIHW.
15. Benedict A, Arellano J, De CE, Baird J. Economic evaluation of duloxetine versus serotonin selective reuptake inhibitors and venlafaxine XR in treating major depressive disorder in Scotland. *J Affect Disord*. 2010;120(1-3):94-104.
16. Black C, Clar C, Henderson R, MacEachern C, McNamee P, Quayyum Z, et al. The clinical effectiveness of glucosamine and chondroitin supplements in slowing or arresting progression of osteoarthritis of the knee: a systematic review and economic evaluation. *Health Technol Assess*. 2009;13(52):1-148.
17. CD, NH. IMS CORE Diabetes Model Briefing Document 4: Utility loss associated with diabetes complications. IMS Health; 2010.
18. Cheng Q, Lazzarini PA, Gibb M, et al. A costeffectiveness analysis of optimal care for diabetic foot ulcers in Australia. *Int Wound J*. 2017;14: 616-628.
19. Clarke PM, Gray AM, Briggs A, et al. A model to estimate the lifetime health outcomes of patients with type 2 diabetes: the United Kingdom Prospective Diabetes Study (UKPDS) Outcomes Model (UKPDS no. 68). *Diabetologia*. 2004;47:1747-59.
20. Coffey JT, Brandle M, Zhou H, Marriott D, Burke R, Tabaei BP, et al. Valuing health-related quality of life in diabetes. *Diabetes Care*. 2002;25(12):2238-43.
21. Colagiuri S, Brnabic A, Gomez M, Fitzgerald B, Buckley A, Colagiuri R. DiabCo$t Australia type 1: assessing the burden of type 1 diabetes in Australia. Canberra, Australia, Diabetes Australia, 2009.
22. Dolan P, Gudex C, Kind P, Williams A. A social tariff for EuroQoL: Results from a UK general population survey. Discussion Paper No. 138. Centre for Health Economics. 1995; University of York (York).
23. Evans M, Khunti K, Mamdani M, et al. Health-related quality of life associated with daytime and nocturnal hypoglycaemic events: a time trade-off survey in five countries. *Health Qual Life Outcomes*. 2013;11(1):90.
24. Goldney RD, Phillips PJ, Fisher LJ, et al. Diabetes, depression, and quality of life: a population study. *Diabetes Care*. 2004;27:1066-70.
25. Hiratsuka Y, Yamada M, Akune Y, et al.; Eye Care Comparative Effectiveness Research Team (ECCERT). Cost-utility analysis of cataract surgery in Japan: a probabilistic Markov modeling study. *Jpn J Ophthalmol*. 2013;57:391-401.
26. Kawasaki R, Akune Y, Hiratsuka Y, Fukuhara S, YamadaM. Cost-utility analysis of screening for diabetic retinopathy in Japan: a probabilistic Markov modeling study. *Ophthal Epidemiol*. 2015;22:4-12.
27. Lee AJ, Morgan CL, Conway P, et al. Characterisation and comparison of health-related quality of life for patients with renal failure. *Curr Med Res Opin*. 2005;21:1777-83.

28. Lee JM, Rhee K, O'Grady MJ, et al. Health utilities for children and adults with type 1 diabetes. *Med Care*. 2011;49(10):924-31.
29. Matza LS, Boye KS, Yurgin N, et al. Utilities and disutilities for type 2 diabetes treatment-related attributes. *Qual Life Res*. 2007;16:1251-65.
30. McEwan P, Poole CD, Tetlow T, et al. Evaluation of the cost-effectiveness of insulin glargine versus NPH insulin for the treatment of type 1 diabetes in the UK. *Curr Med Res* Opin. 2007;23(1 Suppl):S7-S19.
31. McQueen RB, Ellis SL, Maahs DM, et al. Association between glycated hemoglobin and health utility for Type 1 diabetes. *Patient*. 2014;7(2):197-205.
32. Morgan CL, McEwan P, Morrissey M, et al. Characterization and comparison of health-related utility in people with diabetes with various single and multiple vascular complications. *Diabet Med*. 2006;23:1100-5.
33. National Health Service, Department of Health. NHS Reference Costs, 2008-2009. Accessed September 11, 2018. http://webarchive.nationalarchives.gov.uk/20130104223435/http://www.dh.gov.uk/en/Publicationsandstatistics/Publications/PublicationsPolicyAndGuidance/DH_111591. Published 2010.
34. National Institute for Clinical Excellence (NICE). Guidance on the use of long-acting insulin analogues for the treatment of diabetes: insulin glargine [online]. Available from: http://guidance.nice.org.uk/TA53/publicinfo/pdf/English [Accessed 2007 Aug 30].
35. National Institute for Clinical Excellence. Long-acting insulin analogues for the treatment of diabetes - insulin glargine. Accessed August 13, 2018. https://www.nice.org.uk/guidance/ta53/documents/final-appraisaldetermination-longacting-insulin-analogues-for-the-treatment-of-diabetes-insulin-glargine-2.
36. NICE: National Institute for Clinical Excellence. Technology appraisal. Guidance—No. 53. Guidance on the Use of Long-Acting Insuiln Analogues for the Treatment of Diabetes—Insulin Glargine. 12-1-2002.
37. Nyman JA, Barleen NA, Dowd BE, Russell DW, Coons SJ, Sullivan PW. Quality-of-life weights for the US population: self-reported health status and priority health conditions, by demographic characteristics. *Med Care*. 2007: 45: 618.
38. Pratoomsoot C, Smith HT, Kalsekar A, Boye KS, Arellano J, Valen-tine WJ. An estimation of the long-term clinical and economicbenefits of insulin lispro in type 1 diabetes in the UK. *Diabet Med*. 2009;26:803-14.
39. RedekopWK, Stolk EA, Kok E, Lovas K, Kalo Z, Busschbach JJV. Diabetic foot ulcers and amputations: estimates of health utility for use in cost-effectiveness analyses of new treatments. *Diabetes Me*tab. 2004;30:549-56.
40. Smith-Palmer J, Bae JP, Boye KS, Norrbacka K, Hunt B, ValentineWJ. Evaluating health-related quality of life in type 1 diabetes: a systematic literature review of utilities for adults with type 1 diabetes. *Clinicoecon Outcomes Res*. 2016;8:559-571.
41. Sullivan PW, Ghushchyan V. Preference-based EQ-5D index scores for chronic conditions in the United States. *Med Decis Making*. 2006;26(4):410-20.
42. Szabo SM, Beusterien KM, Pleil AM, et al. Patient preferences for diabetic retinopathy health States. *Invest Ophthalmol Vis Sci*. 2010;51:3387-94.
43. Tabaei BP, Shill-Novak J, Brandle M, Burke R, Kaplan RM, Herman WH. Glycemia and the quality of well-being in patients with diabetes. *Qual Life Res*. 2004: 13: 1153.
44. United States Government Accountability Office. End-stage renal disease. Characteristics of kidney transplant recipients, frequency of transplant failures, and cost to Medicare. Report GAO-07-1117, September 2007.
45. Walters N, Gordois A, Brown A, Lindsay P, Gonzalo F, Comas S. Quantifying the Impact of Fear of Hypoglycaemia on Quality of Life. *Value Health*. 2006;9(6):A238.
46. Ward S, Lloyd JM, Pandor A, Holmes M, Ara R, Ryan A, et al. A systematic review and economic evaluation of statins for the prevention of coronary events. *Health Technol Assess.* 2007;11(14):1-iv.
47. Wasserfallen JB, Halabi G, Saudan P, et al. Quality of life on chronic dialysis: comparison between haemodialysis and peritoneal dialysis. *Nephrol Dial Transplant.* 2004;19:1594-1599.
48. Wolowacz S, Pearson I, Shannon P, et al. Development and validation of a cost-utility model for Type 1 diabetes mellitus. *Diabetic Med*. 2015;32(8):1023-35.
49. Yabroff KR, Lawrence WF, Clauser S, Davis WW, Brown ML. Burden of illness in cancer survivors: findings from a population-based national sample. *J Natl Cancer Inst*. 2004;96(17):1322-30.
50. Yeh H-C, Brown TT, Maruthur N, Ranasinghe P, Berger Z, Suh YD, et al. Comparative effectiveness and safety of methods of insulin delivery and glucose monitoring for diabetes mellitus: a systematic review and meta-analysis. *Ann Intern Med*. 2012;157:336-347.

Figure S1. PRISMA Flowchart for SLR and MA Identification, Screening, and Inclusion

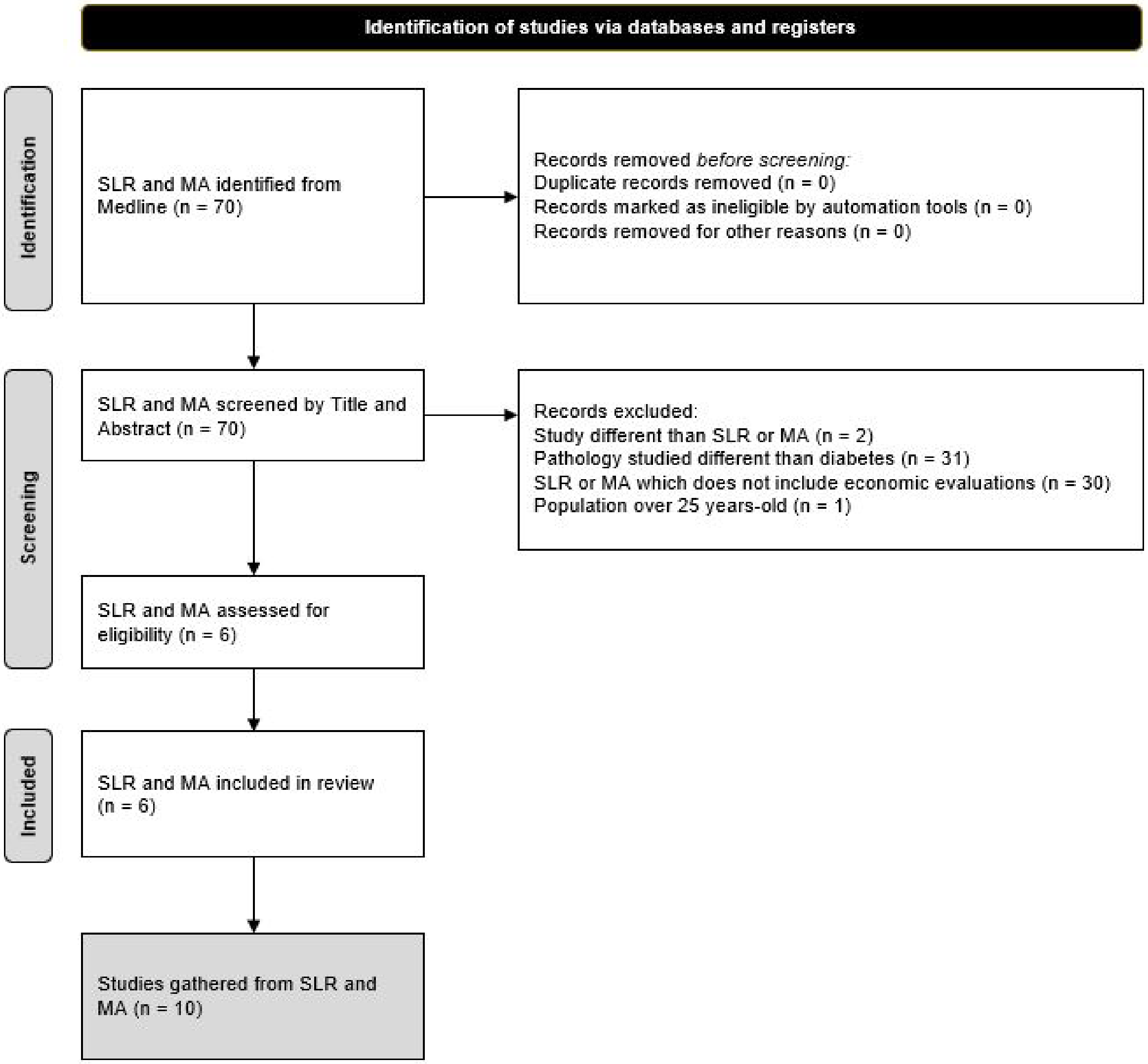

Abbreviations: MA, meta-analysis; SLR, systematic literature review.
